# Multi-kernel support vector regression with improved moth-flame optimization algorithm for software effort estimation

**DOI:** 10.1038/s41598-024-67197-1

**Published:** 2024-07-23

**Authors:** Jing Li, Shengxiang Sun, Li Xie, Chen Zhu, Dubo He

**Affiliations:** Department of Management Engineering and Equipment Economics, Naval University of Engineering, Wuhan, 430033 China

**Keywords:** Moth-flame optimization algorithm, Parameter optimization, Software effort estimation, Support vector regression, Engineering, Mathematics and computing

## Abstract

In this paper, a novel Moth-Flame Optimization (MFO) algorithm, namely MFO algorithm enhanced by Multiple Improvement Strategies (MISMFO) is proposed for solving parameter optimization in Multi-Kernel Support Vector Regressor (MKSVR), and the MISMFO-MKSVR model is further employed to deal with the software effort estimation problems. In MISMFO, the logistic chaotic mapping is applied to increase initial population diversity, while the mutation and flame number phased reduction mechanisms are carried out to improve the search efficiency, as well the adaptive weight adjustment mechanism is used to accelerate convergence and balance exploration and exploitation. The MISMFO model is verified on fifteen benchmark functions and CEC 2020 test set. The results show that the MISMFO has advantages over other meta-heuristic algorithms and MFO variants in terms of convergence speed and accuracy. Additionally, the MISMFO-MKSVR model is tested by simulations on five software effort datasets and the results demonstrate that the proposed model has better performance in software effort estimation problem. The Matlab code of MISMFO can be found at https://github.com/loadstar1997/MISMFO.

## Introduction

In recent years, software applications have become increasingly important in daily life, leading to a growing demand for software projects in various fields. High-reliability, low-cost software products have become crucial in the competitive landscape^1,2^. However, developing successful and reliable software is challenging for any organization, and studies have shown that accurate software effort estimation significantly impacts the success rate of projects^3^. Accurate estimation helps project managers optimize project management, control the development process, and effectively plan and allocate resources. Overestimation may result in wasted resources, while underestimation can also lead to poor product quality or project failure^4^. Therefore, accurate prediction of software effort is an important issue.

To improve the accuracy of software effort estimation, scholars have conducted extensive research and proposed a series of software effort prediction models. These models can be broadly classified into three categories: Expert judgement-based^5–9^, Algorithmic-based^10–12^ and Computational intelligence-based methods^13^. Expert judgment-based models rely on the extensive experience and profound knowledge of relevant personnel, such as project managers, to estimate software project effort. Common expert judgment techniques include the Delphi method and analogy methods. The Delphi method anonymously collects and compiles expert opinions, providing feedback iteratively until a consensus is achieved. Analogy methods estimate the effort required for a new software project by referencing the total effort of previously completed, similar projects. Algorithmic-based models establish the relationship between software effort and its influencing factors by analyzing historical data and refining estimation rules or formulas. Common algorithmic-based models include COCOMO^10–12^, the Use Case Point (UCP) method^14,15^, and the Function Point Analysis (FPA) method^16,17^. The COCOMO model is often used in combination with optimization algorithms such as Genetic Algorithms and Neural Networks^12,18^. The UCP method estimates the effort required for a software project based on the number of use cases in the system, as well as technical and environmental parameters. Determining parameters is a key challenge for the UCP method, and optimization algorithms are typically used to address this issue^14,15^. The FPA method measures the complexity and size of software in terms of function points provided to the user^15^, and subsequently estimates the software effort based on it. A comprehensive review of recent research on FPA is provided by^17^.

Expert judgment-based models rely heavily on the experiences, knowledge, and personal preferences of experts, which can present challenges in identifying experts with relevant knowledge in emerging fields. Furthermore, the expert method demands significant investments of time and resources to gather, discuss, and integrate opinions, making it costly and time-consuming. Additionally, obtaining comprehensive and accurate estimation results for large-scale and complex projects can be difficult with this approach^19^. Conversely, algorithmic-based models depend heavily on historical data, and their accuracy may be compromised when data is insufficient^20^. The estimation results are also sensitive to parameter values, which must be precisely estimated and adjusted. Any deviation in these values can lead to unsatisfactory estimation results. Consequently, researchers are constantly seeking more effective methods to address the problem of software effort estimation^5^.

The development of artificial intelligence technology offers novel solutions to the problem of software effort estimation. Models based on computational intelligence, such as machine learning and deep learning, estimate software effort by leveraging advanced computer techniques and improving accuracy through iterative training^3^. The most commonly used machine learning methods for software effort estimation include artificial neural network (ANN), decision trees, logistic regression, case-based reasoning, SVR, k-nearest neighbors (KNN), linear regression, and Bayesian methods^21^. Machine learning methods are highly dependent on the dataset, with different methods performing variably across different datasets. To enhance the accuracy and stability of these models, integrated learning techniques have been introduced into the field of software effort estimation^22–25^. Among the above methods, SVR has garnered significant attention due to its effectiveness in handling small samples and nonlinear problems. Oliveira compared SVR, neural networks, and linear regression for software effort estimation, concluding that SVR was more effective^26^. Martino et al. optimized SVR using taboo search and applied it to estimate software effort^27^. Hai et al. employed SVR and multivariate linear regression to estimate the duration of software projects, confirming that SVR outperformed in project duration estimation using six evaluation metrics and *t*-tests^28^. Floriano et al.^29^ and Sakhrawi et al.^30^ used SVR for enhanced software project effort estimation. Utkin et al. proposed a new software reliability growth model and utilized SVR to estimate the probability of trouble-free software operation^31^. Nevendra et al. used SVR as the base learner in ensemble learning to estimate software project effort and duration^32^. Pospieszny et al. also used SVR as a base-learner in integrated learning to estimate software project effort and duration^33^. Although SVR has been widely adopted and has achieved some success in software effort estimation, it still faces practical challenges, particularly in kernel function selection and parameter optimization.

The selection of a kernel function poses a significant challenge when using SVR for prediction. The linear kernel is computationally efficient but limited to linearly divisible problems. The polynomial kernel can handle linearly inseparable problems and exhibit global properties, but it requires numerous parameters and suffers from slow computational speeds. The radial basis function (RBF) kernel is effective for nonlinear data and exhibits strong local learning capabilities, but its capacity for global learning is constrained^34^. Therefore, the choice of kernel function should be tailored to the specific characteristics of the data. In practice, due to the diversity of datasets, a single kernel function often fails to accurately interpret the data^35,36^, making single-kernel SVR inadequate for uncovering complex patterns^37^. To better capture the intricate features of the data and avoid the arbitrariness of human selection, a potential solution is to combine the advantages of different kernel functions. This can be achieved by using an optimal combination of multiple kernel functions instead of a single kernel function, thereby improving the prediction performance of SVR^38^. However, this approach entails additional complexity, especially concerning the optimization of hyperparameters.

In both SVR and kernel functions, the determination of hyperparameters is essential, and optimizing these parameters is crucial for Multi-Kernel SVR (MKSVR) as it directly influences the predictive performance of the model. Manual parameter optimization based on experience often fails to achieve the desired results and requires extensive time to make multiple attempts before finding a suitable parameter^38^. The grid search method suffers from high computational complexity and fixed parameter granularity, making it ineffective when the optimal parameter value lies outside the predefined range.

Meta-heuristics offer an effective alternative to exact methods for solving complex real-life optimization problems within polynomial time^39^. Owing to their effectiveness and simplicity in design and implementation, meta-heuristic algorithms are widely applied across various engineering domains^40,41^, particularly in parameter optimization^40^. These algorithms are mostly inspired by creatures’ strategies existing in nature, which is a rich source for inspiration and development of nature-inspired (NI) algorithms. Various algorithms were presented that their source of inspiration is nature^42^. Such as particle swarm optimization (PSO)^43^, differential evolution (DE)^44^, genetic algorithm (GA)^45^, Moth-flame optimization Algorithm (MFO)^46–48^, Grey Wolf optimization Algorithm (GWO)^49^, Harris Hawks Optimization (HHO)^50^, Slime Mould Algorithm (SMA)^51^, Dwarf Mongoose Optimization (DMO)^52^ and etc.

Due to the effectiveness of meta-heuristic algorithms in parameter optimization, they are also employed in optimizing the parameters for MKSVR. Kui et al. utilized the artificial bee colony (ABC) algorithm to optimize hyperparameters in the MKSVR and applied the refined hybrid model to predict LIB capacity degradation^37^. Hua et al. introduced an MKSVRE model using Unified Optimization and whale optimization algorithm (WOA) for parameter selection in wind speed prediction^34^. While these algorithms have partially addressed parameter optimization for the MKSVR model, they still present certain limitations in practical applications. The ABC often suffers from slow convergence and complex parameter settings. The WOA tends to converge prematurely. Although unified optimization are comprehensive, they are computationally complex and struggle to effectively balance exploration and exploitation. The MFO algorithm has received extensive attention from scholars due to its advantages of having few parameters, easy implementation, and its effectiveness as a global search mechanism that can explore and exploit the entire search space^53^. Consequently, it has been widely utilized for practical optimization problems in the past few years, such as chemical, economic applications, image processing, and medical^54^. Although MFO exhibits superior performance in addressing various practical issues, its spiral search mechanism makes the algorithm focus more on local exploitation than global exploration^55^ and it is easy to fall into local optimal solutions during the optimization process^54^.

To overcome these limitations, considerable research efforts have been made in recent years. These advancements have notably enhanced the performance of MFO in terms of convergence speed and exploratory capabilities. However, several constraints still remain that require further resolution. First, the rapid loss of population diversity during the search process remains an unresolved issue. Second, the adaptive adjustment of the search strategy across different phases requires further investigation.

In this study, an improved MFO algorithm called MISMFO is proposed, which aims to optimize the hyperparameters of the MKSVR-based software effort estimation model. By addressing the limitations of traditional MFO variants and incorporating innovative mechanisms, MISMFO seeks to achieve superior optimization performance and enhance the predictive accuracy of the model. The main contributions of this paper are as follows:

1. Logistic chaotic mapping is employed to generate the initial population of moths, which ensures the diversity of the initial population in MISMFO.
2. The flame mutation mechanism is utilized to enhance population diversity during the search process, enabling the algorithm to escape local optima and explore new regions in the solution space. Additionally, the flame number phased reduction mechanism is introduced to improve the search efficiency.
3. An adaptive weight mechanism is implemented to update the positions of the moths, enabling the moths to dynamically adjust their strategy based on the ratio between their fitness and the optimal flame fitness, thus improving the convergence speed and accuracy of the algorithm.
4. The MISMFO algorithm was compared with other optimization algorithms and MFO variants on fifteen benchmark functions and CEC 2020 test set. Additionally, MISMFO was employed to optimize the hyperparameters of MKSVR for estimating software effort. The results verify the effectiveness of the proposed model.

The rest of this paper is organized as follows: section “Related work” briefly introduces the original MFO algorithm and the MKSVR. Section “Methodology” details the MISMFO algorithm and the construction process of the MISMFO-MKSVR model. Section “The proposed method” compares the performance of MISMFO with other optimization algorithms using benchmark functions. Section “Experiment” validates the effectiveness of the MISMFO-MKSVR model on five publicly available software datasets. Section “Case study” summarizes the research findings in a forward-looking manner.

## Related work

Meta-heuristic algorithms have demonstrated their efficacy in addressing complex problems characterized by high dimensionality, multi-modality, and non-differentiability^42^. As a result, they have been widely applied across various fields, such as community detection^56^, engineering cases^57^, System identification^40^, medical diagnosis^58^, image segmentation^59^ and multi-objective optimization^60^. Meta-heuristic algorithms can be generally divided into two categories: non-nature-inspired and nature-inspired (NI) algorithms. Although a few algorithms have been developed in the first category, such as Taboo Search (TS)^61^. The majority of meta-heuristic algorithms are inspired by nature and have been widely applied to optimization problems^62^. These NI algorithms can be further classified into three categories: evolutionary, physics-based, and swarm intelligence algorithms. Evolutionary algorithms (EAs) represent a class of iterative optimization algorithms that emulate the evolutionary processes found in nature. The most well-known EAs are GA and DE. Physics-based algorithms mimic physical rules in nature. Some popular algorithms in this category are black hole (BH)^63^, atom search optimization (ASO)^64^. Swarm intelligence algorithms (SIs) are inspired by the collective behavior of social creatures, based on swarm intelligence and evolution theory, they can generate a set of random solutions to automatically investigate the whole search space through multiple iterations until the optimal solution is found. Some of the advanced and newest algorithms in this category include GWO, MFO, HHO, SMA, DMO. The Moth-flame Optimization (MFO) is a population-based stochastic search algorithm, which was proposed in 2015^65^. In MFO, moths and flames are employed as candidate solutions, where the optimal flame signifies the current optimal solution. Moths adjust their positions through a spiral trajectory in each iteration, while the algorithm iteratively updates the flame position to seek the optimal solution. Owing to its simplicity, minimal control parameters, and ease of implementation, MFO has been widely applied for parameter optimization, etc. Wei et al. considered that the MFO more accurately and converges faster compared with traditional optimization algorithms (e.g. PSO, GA), and used LS-SVM based on the MFO optimization to diagnose the bearing faults^66^. Kalita et al. used MFO to optimize the hyperparameters of SVM in a dynamic environment and verified that the model optimized by MFO had a higher accuracy and better performance in intrusion detection system^67^. Talaat et al. optimized an artificial neural network (ANN) by MFO to improve its arithmetic accuracy^68^.

Since the introduction of MFO, various modifications have been made to overcome its limitations and enhance its performance. Lin et al. introduce an inertia weighting strategy and the Cauchy mutation operator to improve the moth-flame optimization algorithm. The former balances the search and mining capabilities at the population location search equation, and the latter helps to increase the diversity of the masses and to void avoid entrapment into local optima^69^. Wang et al. adopted two chaotic strategies to improve MFO to increase population diversity^70^. Pelusi et al. divided the search process of MFO into three phases, and proposed corresponding search strategies for different phases to balance the relationship between exploration and exploitation^71^. Sahoo et al. proposed an improved moth flame optimization (MFO) algorithm based on dynamic opposite learning (DOL), incorporating a modified DOL strategy to effectively address the issues of premature convergence and convergence to local optima of the basic MFO^72^. In subsequent research, they further developed two enhanced variants of MFO, specifically designed for multi-objective optimization problems and COVID-19 CT image segmentation^59,60^, respectively. Wang et al. employed inertia weights, uniform initialization and spiral curve updating mechanism to enhance the global search capability of MFO^73^. Shan et al. proposed a double adaptive weighting mechanism, which enables the algorithm to adaptively adjust the search strategy in different periods, thus achieving the flexible conversion between the exploration and exploitation^58^. Zhao et al. investigated the effects of mutation and chaotic mechanism on MFO, and applied the improved MFO to real optimization problems^54,74–76^. Elaziz et al. applied the opposition-based learning technique to generate the optimal initial population, meanwhile, the differential evolution was used to improve the exploitation ability of the MFO^77^. Sharma et al. employed an opposition-based learning mechanism to initialize the search population for enhancing exploration, as well utilized levy flight distribution to avoid the stagnation of solutions in local optima^47^. Nguyen et al. hybridized levy flight and logarithmic functions for updating the flame to improve the optimization performance of the MFO^78^. Jia et al. used the adaptive inertia weighting mechanism to enhance the exploration and development of the algorithm and improved MFO by combining it with practical problems to achieve better results^79^.

## Methodology

### Moth-flame optimization algorithm

The MFO algorithm is a novel swarm optimization algorithm proposed by Mirjalili^65^, which is inspired by the nocturnal navigation behaviour of moths and the MFO algorithm is based on mathematical modelling of moths, flames and the positional relationship between them. The search space of the moth is the solution space of the target problem, and the position of the moth in the search space is the problem variable. In order to find a better solution, the moth will performs a spiral search around its corresponding flame and update its position. The flame is the optimal position obtained by the moth so far. The position of the moth can be represented by

1 $$\begin{aligned} {\textbf {M}}=\left[ \begin{array}{c} M_1\\ M_2\\ \vdots \\ M_n\\ \end{array} \right] =\left[ \begin{matrix} m_{11}&{} \quad m_{12}&{} \quad \cdots &{}\quad \cdots &{}\quad m_{1d}\\ m_{21}&{} \quad m_{22}&{}\quad \cdots &{}\quad \cdots &{} \quad m_{2d}\\ \vdots &{}\quad \vdots &{}\quad \vdots &{}\quad \vdots &{}\quad \vdots \\ m_{n1}&{} \quad m_{n2}&{}\quad \cdots &{} \quad \cdots &{} \quad m_{nd}\\ \end{matrix} \right] , \end{aligned}$$

where *n* is the number of moths and *d* is the dimension of the search space. The fitness value of each moth can be calculated by the fitness function and stored in the vector ${\textbf {O}} {\textbf {M}}$ as

2 $$\begin{aligned} {\textbf {O}}{} {\textbf {M}} =\left[ \begin{matrix} OM_1&{}\quad OM_2&{} \quad \cdots &{} \quad OM_n\\ \end{matrix} \right] , \end{aligned}$$

Since each moth has a flame corresponding to it, so the flame matrix has a similar structure to the moth matrix, which can be defined by the matrix ${\textbf {F}}$ in Eq. (3), and its fitness value is shown in Eq. (4).

3 $$\begin{aligned} {\textbf {F}}=\left[ \begin{array}{c} F_1\\ F_2\\ \vdots \\ F_n\\ \end{array} \right] =\left[ \begin{matrix} f_{11}&{}\quad f_{12}&{}\quad \cdots &{}\quad \cdots &{}\quad f_{1d}\\ f_{21}&{} \quad f_{22}&{} \quad \cdots &{} \quad \cdots &{} \quad f_{2d}\\ \vdots &{} \quad \vdots &{} \quad \vdots &{} \quad \vdots &{} \quad \vdots \\ f_{n1}&{}\quad f_{n2}&{} \quad \cdots &{} \quad \cdots &{}\quad f_{nd}\\ \end{matrix} \right] , \end{aligned}$$

4 $$\begin{aligned} {\textbf {O}}{} {\textbf {F}} = \left[ \begin{matrix} OF_1&{} OF_2&{} \cdots &{} OF\\ \end{matrix}_n \right] , \end{aligned}$$

The initial position of the moth population is determined by

5 $$\begin{aligned} m_{ij}=lb_j+r(ub_j-lb_j), \end{aligned}$$

where $m_{ij}$ denotes the position of the *i*-th moth in the *j*-th dimension. $lb_j$ and $ub_j$ denote the lower and upper bounds of the *j*-th dimension of the search space, respectively. Denote ${\textbf {l}}{} {\textbf {b}} = [lb_1,lb_2,\ldots ,lb_d]$, ${\textbf {u}}{} {\textbf {b}}= [ub_1,ub_2,\ldots ,ub_d]$ and *r* is a random value between 0 and 1.

During the search process, when the moths spiral around the flames to locate the global optimum, each moth corresponds to a flame for position update. However, the moths updating their positions relative to *n* flames may reduce the efficiency of seeking the optimal region, and in order to balance the exploration and exploitation, the number of flames is dynamically reduced in the search process according to the following equation

6 $$\begin{aligned} f_{no}=round\left( n-\frac{t(n-1)}{T}\right) , \end{aligned}$$

where $f_{no}$ is the number of flames, *t* and *T* are the current and maximum number of iterations, respectively, and *round* is the rounding function. As the number of flames decreases, there are some changes in the correspondence between moths and flames, with the first $f_{no}$-th moths corresponding to the $f_{no}$ flames, and the subsequent moths corresponding to the $f_{no}$-th flame. The position of moths is updated during the search process according to the following equation:

7 $$\begin{aligned}{} & {} M_{i}^{t+1}=\left\{ \begin{array}{c} D_{i}^{t}\cdot e^{bl}\cdot \cos 2\pi l+F_{i}^{t}, i\le f_{no}\\ D_{i}^{t}\cdot e^{bl}\cdot \cos 2\pi l+F_{f_{no}}^{t}, i>f_{no}\\ \end{array} \right. \end{aligned}$$

8 $$\begin{aligned}{} & {} D_i=\left\{ \begin{array}{c} \left| M_i-F_i \right| ,i\le f_{no}\\ \left| M_i-F_{f_{no}} \right| ,i>f_{no}\\ \end{array} \right. \end{aligned}$$

where $D_i^t$ denotes the Euclidean distance between the *i*-th moth $M_i$ and its corresponding flame $F_i$ at the *t*-th iteration. *b* is a constant defining the shape of the spiral curve. *l* is a random number between [*r*, 1], and $r=-1+t(-{{1}/{T}})$. The *r* value decreases linearly from $-1$ to $-2$ with the iteration process. Until the maximum number of iterations is reached, the position of the first flame and the fitness value are returned, which is the optimal solution, and the MFO algorithm end.

### Multiple-kernel SVR

SVR is a non-linear kernel-based regression method which tries to find a regression hyper-plane with small rick in high-dimensional feature space. Among the various types of SVR, the most commonly used is $\varepsilon$-SVR which introduces an $\varepsilon$-insensitive loss function. Given a set of samples $\left\{ \left( {\textbf {x}}_i,y_i \right) \right\} _{i=1}^{l}$, where $\textbf{x}_i\in \mathbb {R}^n$ and $y_i\in \mathbb {R}$, and *l* is the number of samples. The objective function of $\varepsilon$-SVR is defined as

9 $$\begin{aligned} &\min _{\textbf{w},b} \frac{1}{2}\langle \textbf{w},\textbf{w}\rangle +C\sum _{i=1}^l(\xi _i+\hat{\xi }_i), \\ \text {s.t.}&(\langle \textbf{w},\phi (\textbf{x}_i)\rangle +b)-{y}_i\leqslant \varepsilon +\xi _i, \\&y_{i}-(\langle \textbf{w},\phi (\textbf{x}_{i})\rangle +\varvec{b}) \leqslant \varepsilon +\hat{\xi }_{i}, \\&\xi _{i},\hat{\xi }_{i}\geqslant 0,\quad i=1,\ldots ,l, \end{aligned}$$

where *C* is a parameter which gives a trade-off between model complexity and training error, $\xi _i$ and $\hat{\xi }_i$ are slack variables for exceeding or being below the output value by more than $\varepsilon$, respectively. Denote $\phi :X\rightarrow \mathscr {F}$ as a nonlinear mapping function from the input space to a feature space $\mathscr {F}$. The regression hyperplane in $\mathscr {F}$ is derived as

10 $$\begin{aligned} f(\textbf{x})=\langle \textbf{w},\phi (\textbf{x})\rangle +{b}, \end{aligned}$$

where $\textbf{w}$ and *b* are regression coefficients and bias, respectively. To solve Eq. (9), the Lagrange function is introduced and Eq. (9) is transformed into the following dual form

11 $$\begin{aligned} \max _{\varvec{\alpha },\varvec{\hat{\alpha }}}&\quad \sum _{i=1}^ly_i(\hat{\alpha }_i-\alpha _i)-\hat{\varepsilon }\sum _{i=1}^l(\hat{\alpha }_i+\alpha _i)\\&\quad -\frac{1}{2}\sum _{i=1}^l\sum _{j=1}^l(\hat{\alpha }_i-\alpha _i)(\hat{\alpha }_j-\alpha _j)\varvec{K}(\textbf{x}_i,\textbf{x}_j),\\ \mathrm {s.t.}&\quad \sum _{i=1}^l(\hat{\alpha }_i-\alpha _i)={0},\\&\quad {C}\geqslant \alpha _i,\hat{\alpha }_i\geqslant 0,\quad i=1,\ldots ,l, \end{aligned}$$

where ${\alpha }_i$ and $\hat{\alpha }_i, i=1,\ldots , l$ are Lagrange multipliers, and $\mathbf {\alpha } = [\alpha _1,\alpha _2,\ldots ,\alpha _l]$ and $\hat{\mathbf {\alpha }} = [\hat{\alpha }_1,\hat{\alpha }_2,\ldots ,\hat{\alpha }_l]$. The $K(\textbf{x}_i,\textbf{x}_j)$ is a kernel function which represents the inner product $\left<\phi (\textbf{x}_i),\phi (\textbf{x}_j)\right>$.

The Eq. (11) can be solved by SMO. Suppose $\alpha _i^*$ and $\hat{\alpha }_i^*, i =1,\ldots ,l$ are the optimal values obtained. The final regression function is then given by:

12 $$\begin{aligned} f(\textbf{x})=\sum _{i=1}^l\left( \hat{\alpha }_i^*-\alpha _i^*\right) K(\textbf{x}_i,\textbf{x})+b^*, \end{aligned}$$

where $b^*=y_k+\varepsilon -\sum _{i=1}^l\left( \hat{\alpha }_i^*-\alpha _i^*\right) K(\textbf{x}_i,\textbf{x}_k)$ is obtained from any $\alpha _i^*$ with $0<\alpha _i^*<C$.

The traditional SVR method uses a single mapping function $\phi$ and therefore yields a single kernel function *K*. However, when a dataset has a locally varying distribution, using a single kernel may not capture the varying distribution well^80^. Multiple-Kernel Learning (MKL) can help address this issue, MKL combines several mapping functions to do aggregate mapping. A simple direct sum fusion applies a vector of *M* mapping function^80^, i.e.,

13 $$\begin{aligned} \Phi (\textbf{x}) =[\phi _1(\textbf{x})\phi _2(\textbf{x}), \ldots , \phi _m(\textbf{x})], \end{aligned}$$

to map the input space to the feature space. The weighted sum fusion is adopt with the following mapping function:

14 $$\begin{aligned} \Phi (\textbf{x})= \begin{bmatrix} \sqrt{\lambda _1}\phi _1(\textbf{x}),\sqrt{\lambda _2}\phi _2(\textbf{x}),\ldots ,\sqrt{\lambda _m}\phi _m(\textbf{x}) \end{bmatrix}, \end{aligned}$$

where $\lambda _1, \lambda _2, \ldots , \lambda _m$ are weights of component functions. Denote the weight vector $\varvec{\lambda }=\left[ \lambda _1,\lambda _2,\ldots ,\lambda _m \right]$. The objective function of multiple-kernel SVR is as follows,

15 $$\begin{aligned} \min _{\textbf{w},b,\varvec{\lambda }}&\frac{1}{2}\langle \textbf{w},\textbf{w}\rangle +C\sum _{i=1}^l{(}\xi _i+\hat{\xi }_i),\\ \textrm{s}.\textrm{t}.&(\langle \textbf{w},\Phi (\textbf{x}_i)\rangle +b)-y_i\leqslant \varepsilon +\xi _i,\\&y_i-(\langle \textbf{w},\Phi (\textbf{x}_i)\rangle +b)\leqslant \varepsilon +\hat{\xi }_i,\\&\xi _i,\hat{\xi }_i\geqslant 0,\quad i=1,\ldots ,l,\\&\,\, \lambda _m\geqslant 0,\quad m=1,\ldots ,M,\\&\sum _{m=1}^M{\lambda _m}=1.\\ \end{aligned}$$

where $\Phi$ is the vector of function mappings of Eq. (14). Likewise, by introducing the Lagrangian as usual, the Eq. (15) can be converted to the following dual form,

16 $$\begin{aligned} \max _{\varvec{\alpha },\hat{\varvec{\alpha }},\varvec{\lambda }}&\sum _{i=1}^l{y_i}(\hat{\alpha }_i-\alpha _i)-\varepsilon \sum _{i=1}^l{(}\hat{\alpha }_i+\alpha _i)\\&-\frac{1}{2}\sum _{i=1}^l{\sum _{j=1}^l{(}}\hat{\alpha }_i-\alpha _i)(\hat{\alpha }_j-\alpha _j)\widetilde{K}(\textbf{x}_i,\textbf{x}_j)\\ \textrm{s}.\textrm{t}.&\sum _{i=1}^l{(}\hat{\alpha }_i-\alpha _i)=0,\\&C\geqslant \alpha _i,\hat{\alpha }_i\geqslant 0,\quad i=1,\ldots ,l,\\&\lambda _m\geqslant 0,\quad m=1,\ldots ,M,\\&\sum _{m=1}^M{\lambda _m}=1.\\ \end{aligned}$$

where

17 $$\begin{aligned} \widetilde{K}(\textbf{x}_i,\textbf{x}_j)&=\langle \Phi \left( \textbf{x}_i \right) ,\Phi \left( \textbf{x}_j \right) \rangle \\&=\lambda _1\langle \phi _1\left( \textbf{x}_i \right) ,\phi _1\left( \textbf{x}_j \right) \rangle +\lambda _2\langle \phi _2\left( \textbf{x}_i \right) ,\phi _2\left( \textbf{x}_j \right) \rangle \\&\quad +\cdots +\lambda _M\langle \phi _M\left( \textbf{x}_i \right) ,\phi _M\left( \textbf{x}_j \right) \rangle \\&=\lambda _1K_1\left( \textbf{x}_i,\textbf{x}_j \right) +\lambda _2K_2\left( \textbf{x}_i,\textbf{x}_j \right) \\&\quad +\cdots +\lambda _MK_M\left( \textbf{x}_i,\textbf{x}_j \right) \\&=\sum _{m=1}^M{K_m\left( \textbf{x}_i,\textbf{x}_j \right) } \end{aligned}$$

is a weighted sum of *M* kernel function $K_1$, $K_2, \ldots , K_M$, corresponding to mapping functions $\phi _1$, $\phi _2, \ldots , \phi _M$, respectively. Now, if we find $\varvec{\lambda },\varvec{\alpha }$ and $\hat{\varvec{\alpha }}$ by solving Eq. (16), the regression function of MKSVR is

18 $$\begin{aligned} f(\textbf{x})=\sum _{i=1}^l\left( \hat{\alpha }_i^*-\alpha _i^*\right) \widetilde{K}(\textbf{x}_i,\textbf{x})+b^*, \end{aligned}$$

where $b^*=y_k+\varepsilon -\sum _{i=1}^l\left( \hat{\alpha }_i^*-\alpha _i^*\right) \widetilde{K}(\textbf{x}_i,\textbf{x}_k)$ is obtained from any $\alpha _k^*$ with $0<\alpha _k^*<C$.

## The proposed method

While the MFO algorithm boasts advantages such as rapid convergence and a simple structure, it faces challenges in balancing exploration and exploitation. Additionally, the population diversity decreases too rapidly during the iterative process, making the algorithm easy to fall into the local optimum. To solve the above problems, a MFO algorithm with multiple improvement strategies called MISMFO is proposed. In MISMFO, firstly, the moth population is initialized through the logistic chaotic mapping mechanism,which ensures the diversity of the initial population. Secondly,the mutation and flame number phased reduction mechanisms are proposed to increase the population diversity during the iteration process, which improves the probability of the algorithm jumping out of the local optimum and enhances the exploration. Finally,a novel adaptive weight mechanism is employed to update the moth positions, which accelerates convergence and improves the accuracy of the algorithm, as well balance the exploration and exploitation.

### Population initialization based on logistic chaotic mapping

**Figure 1:**
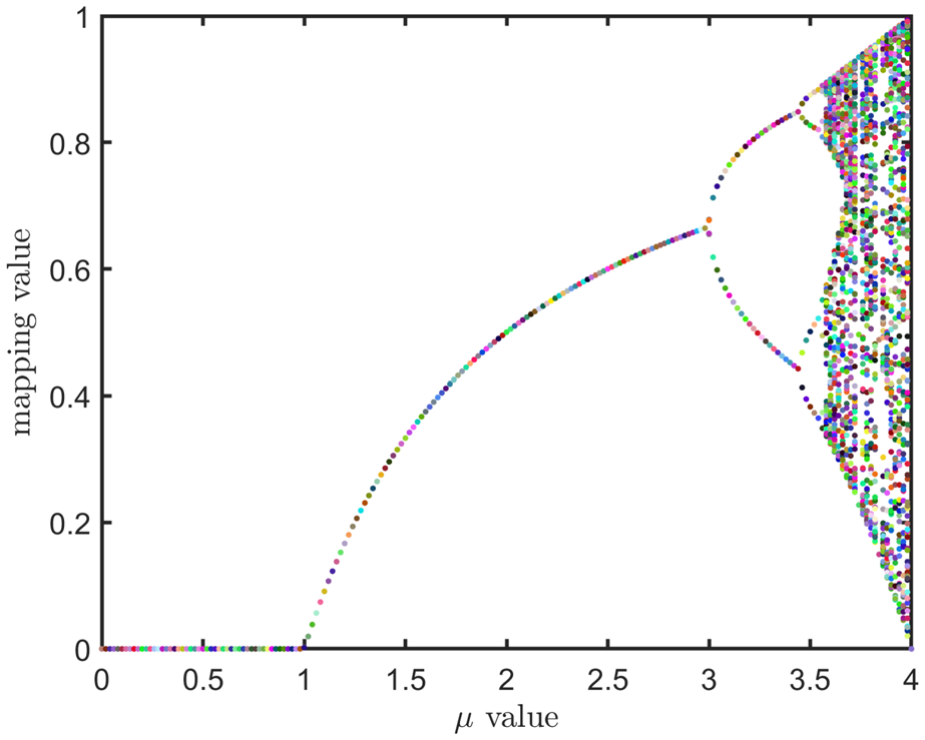
The bifurcation diagram of the logistic model with different values of $\mu$.

The diversity of the initialized population significantly influences the performance of swarm optimization algorithms. A more diverse initial population enhances optimization performance and accelerates convergence. The traditional MFO use a random function based on a uniform distribution to generate the initial solutions. To create a more diverse population, this paper introduces a chaotic mechanism to initialize the moth population. Chaotic techniques are effective in enhancing algorithmic convergence rates and preventing premature convergence to local optima^81,82^. In particular, chaotic sequences surpass probability-based random sequences in improving the traceability and stochasticity of the initial population, thereby increasing its diversity^74^. Among these, logistic chaotic mapping is particularly advantageous due to its simplicity, efficiency, and ability to generate highly diverse initial populations^83^. In this paper, the logistic chaotic mapping is used to initialize the moth population with the following mapping formula:

19 $$\begin{aligned} L_{c+1}=\mu L_c(1-L_c) \end{aligned}$$

where $L_c$ is the *c*-th chaotic variable. $\mu \in \left[ 0,4 \right]$ is the bifurcation parameter indicating the level of chaotic. Figure 1 show the variation in logistic chaotic mapping values for different values of $\mu$. It is evident that the performance of the logistic chaotic map is significantly influenced by the bifurcation parameter. When $\mu = 4$, the system will be transformed into a state of total chaotic, and the initial population position based on Logistic chaotic mapping is calculated as follows:

20 $$\begin{aligned} m_{ij}=lb_j+L_j(ub_j-lb_j) \end{aligned}$$

where $L_j$ denotes the random number generated by the logistic chaotic mapping on the *j*-th dimension.

### Flame update based on mutation mechanisms

In the traditional MFO, the flame is determined by the moths sorted according to their fitness values. When the number of iterations is 1, the flames are obtained by sorting the initial population. When the number of iterations is greater than 1, the moths of the current iteration and the previous iteration are sorted, and the top *n* moths are chosen to obtain the flames. This interaction pattern allows rapid information transfer between the moth population and the flame population. When a single moth falls into the local optimum, it can quickly jump out of the local optimum with the help of flames, but when most moths fall into the local optimum, the algorithm may get trapped in the local optima. To avoid early stagnation of the algorithm, this paper proposes a flame updating mechanism inspired by genetic algorithm mutation. This mechanism uses perturbation and mutation factors to disturb the original flame, enhancing population diversity during the search process and improving the ability to escape from local optimal solutions. Furthermore, since the flame with higher fitness in the population often contain valuable information about the optimal solutions, only the flames with worse fitness are selected for mutation. The mutation mechanism is explained in detail below.

1. **Determine the mutant flames and its number in each iteration.** In order to retain the valuable information of the optimal solution, the mutation is mainly performed for the $n_{mu}$ flames which ranks lower in the original flame ${\textbf {F}}{} {\textbf {I}}= \left[ F_1,F_2,\ldots ,F_n \right]$. Due to MFO algorithm prioritize the exploration in the early search phase and convert to exploitation in the last search phases, the number of mutant flames should change accordingly. For this reason, a perturbation factor *rd* is introduced to control the number of mutant flames.
  21 $$\begin{aligned} rd=R\left( 1-\frac{t}{T}\right) , \end{aligned}$$
  where $R\in [0,1]$ is the maximum perturbation proportion. Then the number of mutant flames $n_{mu}$ is calculated as follows,
  22 $$\begin{aligned} n_{mu\left( t \right) }=round\left( rd_t\cdot n\right) \end{aligned}$$
  where $n_{mu\left( t \right) }$ is the number of mutant flames at the *t*-th iteration, and $rd_t$ is the value of the perturbation factor at the *t*-th iteration, i.e., the proportion of mutation of the original flame at the *t*-th iteration. Thus, the flame to be mutated can be obtained as follows,
  23 $$\begin{aligned} {\textbf {F}}{} {\textbf {M}}=\left[ \begin{array}{c}FM_1\\ FM_2\\ \vdots \\ FM_{n_{mu}} \end{array}\right] = \left[ \begin{array}{cccc}fm_{11}&{}fm_{12}&{}\cdots &{}fm_{1d}\\ fm_{21}&{}fm_{22}&{}\cdots &{}fm_{2d}\\ \vdots &{}\vdots &{}\vdots &{}\vdots \\ fm_{n_{mu}1}&{}fm_{n_{mu}2}&{}\cdots &{}fm_{n_{mu}d}\end{array}\right] \end{aligned}$$
2. **Mutation operations on mutant flame** ${\textbf {F}}{} {\textbf {M}}$. The moth population should explore the whole search space as much as possible in the early phase, while focus on exploit the region near the optimal solution. Therefore, The mutation degree of the flame should be greater in the early phase and gradually decrease over time. To control the mutation degree of the flames, the flame mutation factor *fg* is introduced, which is calculated as follows
  24 $$\begin{aligned} fg=r^\prime \left( 1-\frac{t}{T}\right) ^2 \end{aligned}$$
  where $r^\prime \in [0,1]$ is a random parameter. Obviously, *fg* decreases as *t* increases. The mutation operation for $n_{mu}$ flames in ${\textbf {F}}{} {\textbf {M}}$ is as follows,
  25 $$\begin{aligned} fm_{ij}=\left\{ \begin{array}{c} fm_{ij}+(ub_j-fm_{ij})\times fg_t,r>0.5\\ fm_{ij}-(fm_{ij}-lb_j)\times fg_t,r\le 0.5\\ \end{array} \right. \end{aligned}$$
  where $fm_{ij}$ denotes the value of the *i*-th mutated flame in the *j*-th dimension, $r\in [0,1]$ is random parameter. The mutated flame ${\textbf {F}}{} {\textbf {M}}^\prime$ is obtained.
3. **Comparison and ranking based on the fitness value**. Compare and rank the fitness values of the original flame ${\textbf {F}}{} {\textbf {I}}$ and the mutant flame ${\textbf {F}}{} {\textbf {M}}^\prime$, and select the top *n* flames to participate in the update calculation of the moth position in this round of iteration.

### Flame number phased reduction mechanism

The update of the moth positions is guided by the flame. According to Eq. (6), the number of flames linearly decreases during the search process, causing the search space for moths to gradually narrow down. The population diversity is lost rapidly in the optimization process, thus the MFO algorithm may fall into the local optimum and converge prematurely. To improve the search efficiency of the algorithm, this paper proposes a flame number phased reduction mechanism that divides the whole search process into three phases: (I) In the first phase, the moths focus on the exploration, since the number of flames should decrease slowly to enhance the moths’ ability to explore the entire search space. (II) The second phase is the transition phase, the moths gradually shift its focus from exploration to searching for the optimal region, thus the number of flames should decrease steadily to improve the search efficiency. (III) In the third phase, the moths concentrate on the exploitation. To accelerate the convergence of the algorithm, only the optimal subset of flames is selected to update the moths’ positions. Therefore, the number of flames should decrease rapidly. For this purpose, phase division factors $\delta _1$ and $\delta _2$ are introduced to divide the search process, and the specific division calculation is as follows,

26 $$\begin{aligned} \left\{ \begin{array}{c} P_1=round(\delta _1\times T)\\ P_2=round(\delta _2\times T)\\ \end{array} \right. \end{aligned}$$

where $P_1$ is the number of iterations in the first phase, $P_2$ is the number of iterations in the first two phases. $\delta _1, \delta _2 \in [0,1]$, where $\delta _1$ is the division factors for the first and second phases, while $\delta _2$ is the division factors for the second and third phases. The whole search process will be divided into three phases: $\left[ 1,P_1 \right]$, $\left[ P_1,P_2 \right]$ and $\left[ P_2,T \right]$, then the number of flames reduced in different phase is calculated as follows,

27 $$\begin{aligned} fp_{no}\!\!=\!\!{\left\{ \begin{array}{ll} round\!\left( n-\frac{n-1}{(T\times {P_1}^4)}t^5 \right) \!,1\le t<P_1\\ round\!\left( n-\frac{t(n-1)}{T} \right) \!,P_1\le t\le P_2\\ round\!\left( \frac{\left( n-1 \right) T+P_2(1-n)}{(P_2-T)^4T}(t-T)^4+1 \right) \!, P_2<t\le T\\ \end{array}\right. } \end{aligned}$$

**Figure 2:**
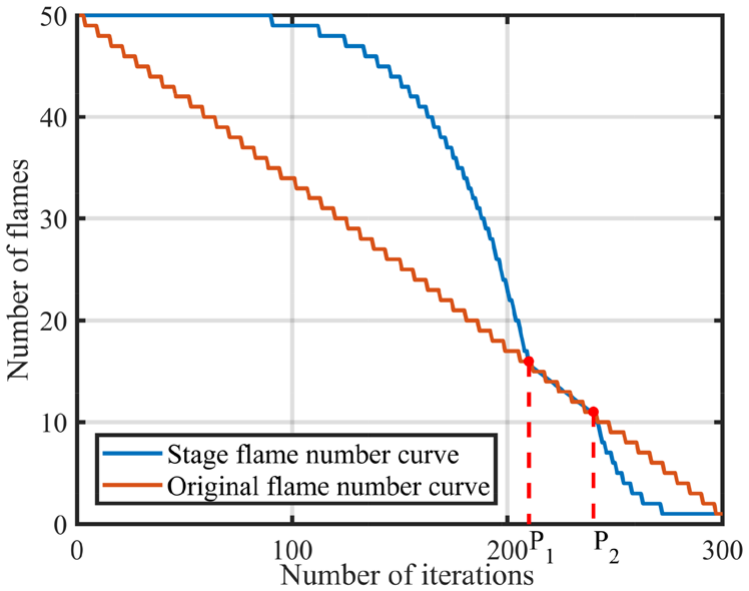
Diagram of the flame number in different stages.

When the population size $n=50$, maximum number of iterations $T=300$ , $\delta _1= 0.7$ and $\delta _2=0.8$. The number of flames decreases according to different phases is shown in Fig. 2.

### Moths positions update based on adaptive weight

In the traditional MFO, the moths search for the optimum in a spiral way around the flames, the update of the moths positions is exclusively influenced by the corresponding flames, and the moths follow a fixed spiral curve as their optimal path during the search process, which makes the moths easy to fall into the local optimum. Hence, to improve the accuracy of the algorithm, this paper introduces the optimal flame to participate in moths positions update along with their corresponding flames, and employs adaptive weight to regulate the influence of the two flames on moths positions update. In the exploration phase, i.e., $[ 1, P_1]$, the moths should scour the entire search space freely to identify the potential optimal solution regions, thus their positions update should not be influenced by the optimal flames and use Eq. (7) for the position update calculation.

In the transition and exploitation phase, i.e. $\left[ P_1,T \right]$, the moths should gradually approach the potential region of the optimal solution and search around it. To accelerate the convergence and prevent the moths from getting trapped in local optima during the approach process, the optimal flame is introduced to participate in the moths positions update. Hence, the moths positions update mechanism in this phase is as follows

28 $$\begin{aligned} M_{i}^{t+1}={\left\{ \begin{array}{ll} D_{i}^{t}e^{bl}\cos 2\pi l+\omega _{i}F_{i}^{t}+(1-\omega _{i})F_{best}^t, i\le fp_{no}\\ D_{i}^{t}e^{bl}\cos 2\pi l+\omega _{i}F_{fp_{no}}^{t}+(1-\omega _{i})F_{best}^t,i>fp_{no}\\ \end{array}\right. } \end{aligned}$$

where $F_{i}^{t}$ denotes the *i*-th flame generated at the *t*-th iteration, $F_{best}^t$ represents the optimal flame obtained so far, and $\omega _{i}$ denotes the adaptive weight of the *i*-th moth at the *t*-th iteration. The $\omega _{i}$ is mainly determined by the fitness of the current moth and the optimal flame, which is calculated as follows,

29 $$\begin{aligned} \omega _i =\frac{1}{2}\sin \left( -\frac{\pi }{2}+\pi \left| \frac{{\textbf {O}}{} {\textbf {F}}_{best}}{{\textbf {O}}{} {\textbf {M}}_i} \right| \right) +\frac{1}{2} \end{aligned}$$

where ${\textbf {O}}{} {\textbf {M}}_i$ is the fitness value of the *i*-th moth, ${\textbf {O}}{} {\textbf {F}}_{best}$ is the fitness value of the optimal flame.

When the current moth is far from the optimal position then the $\omega _i$ tends to 0, the next moth position is updated most in relation to the optimal flame and less in relation to its corresponding flame, which helps the moths escape from the local optima and quickly approach the region of the optimal solution. Therefore, it can accelerate the convergence of the algorithm. Conversely, when the current moth is located near the optimal position, the $\omega _i$ tends to 1. It means that the current moth is updated mainly with respect to its corresponding flame, which allows the moth to conduct a thorough search near the optimal solution region, thereby improving the accuracy of the algorithm. Finally, the pseudo-code for the proposed MISMFO algorithm is given in Algorithm 1.

**Algorithm 1:**
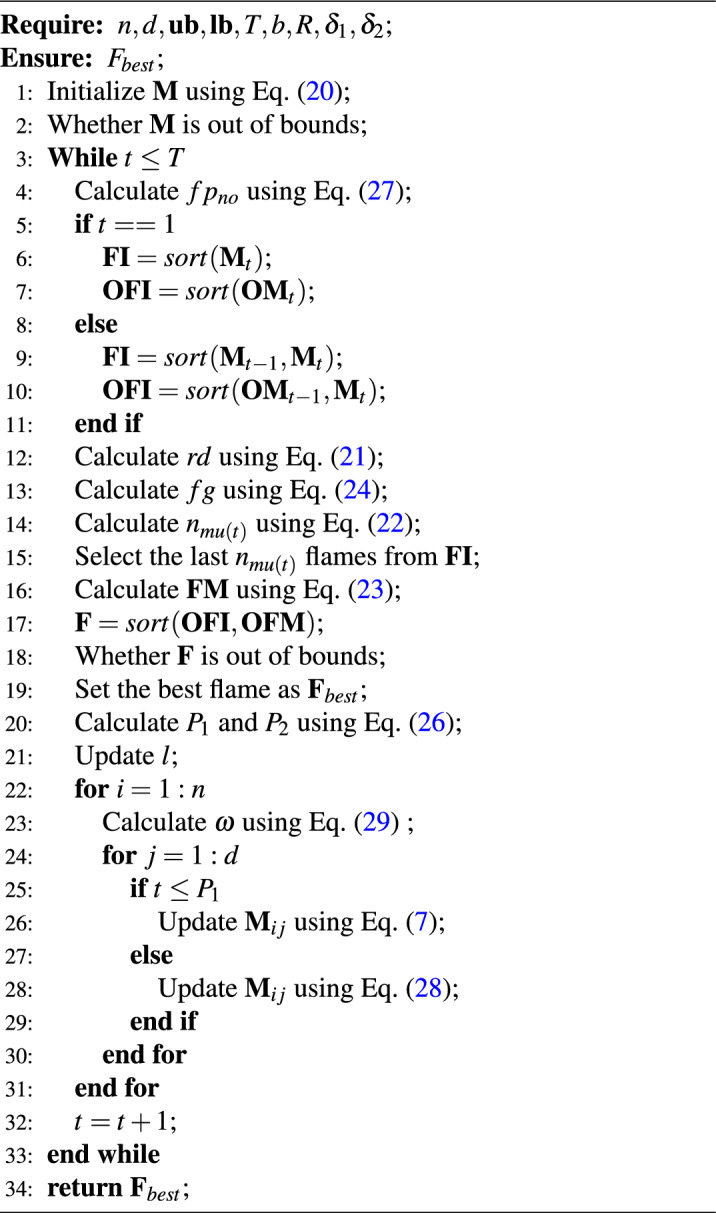
Pseudo-code of MISMFO

### Kernel function selection and the process of model construction

**Figure 3:**
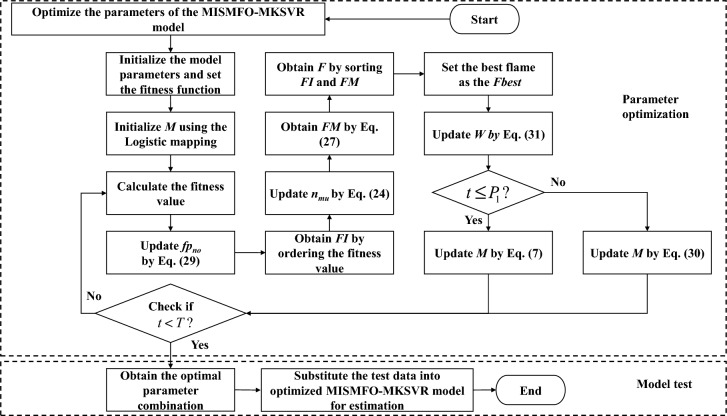
Flow diagram of MISMFO optimisation for MKSVR.

Since the performance of MKSVR is greatly affected by the hyperparameters and kernel parameters, the proposed MISMFO algorithm is used to optimize the weights of kernel function, hyperparameters and kernel parameters in MKSVR. The common kernel functions include linear kernel, Gaussian kernel, polynomial kernel, and Sigmoid kernel. Among them, the Gaussian kernel is a typical local kernel function with strong local generalization ability, while the polynomial kernel is a typical global kernel function that can better capture nonlinear relationships in data and has efficient computation. In this paper, a linear combination of Gaussian and polynomial kernels was employed as a multi-kernel function. The Gaussian kernel is defined as follows:

30 $$\begin{aligned} K_{\sigma }(\textbf{x}_i,\textbf{x}_j):= \langle \phi (\textbf{x}_i),\phi (\textbf{x}_j)\rangle =\exp \left( -\frac{\Vert \textbf{x}_i-\textbf{x}_j\Vert ^2}{\sigma ^2} \right) , \end{aligned}$$

where $\sigma ^2$ is the variance of the Gaussian kernel, while the polynomial kernel function is defined as

31 $$\begin{aligned} K_{d}\left( \textbf{x}_i,\textbf{x}_j \right) :=\left( \gamma \ \textbf{x}^{\top }\textbf{x}+c \right) ^d \end{aligned}$$

where $\gamma >0$ is the slope, *c* is the intercept and *d* is the power parameter. Hence, the kernel function used in this paper can be defined as follows:

32 $$\begin{aligned} K\left( \textbf{x}_i,\textbf{x}_j \right) =w_1K_{\sigma }\left( \textbf{x}_i,\textbf{x}_j \right) +w_2K_d\left( \textbf{x}_i,\textbf{x}_j \right) \end{aligned}$$

where the $w_1$ is the weight of Gaussian kernel function, and $w_2$ is the weight of polynomial kernel function. The flow of model establishment is shown in the Fig. 3.

### The complexity of the MISMFO algorithm

The big-*O* notation is used for the time and space complexity of MISMFO algorithm. The time complexity of the proposed MISMFO depends on the initialization of moth position($T_{IMP}$), mutation operation ($T_{MO}$), distance calculation ($T_{DC}$), fitness calculation of moth position ($T_{FCMP}$) and flame generation ($T_{FG}$). Let maximum iterate number, variable number, and moths’ number, the time complexity of the fitness function are denoted by *T*, *D*, *N* and *L*, respectively. Here we will use time complexity for the comparison of both MISMFO and MFO algorithms. Computational complexity for sorting *N*-flame and *N*-moth are lying between $O \left( T*2N*log(2N)\right)$ and $O \left( T*(2N)^2\right)$ towards worst and best case. The overall time complexity of the proposed MISMFO ($T_{MISMFO}$) for the worst case is as follows,

33 $$\begin{aligned} T_{MISMFO}&= T_{IMP} + T_{MO} + T_{DC} + T_{FCMP} + T_{FG} \\&= O\left( N*D \right) + O\left( T*N*D \right) + O\left( T*N*D \right) + O\left( T*N*D \right) + O\left( T*(2N)^2\right) \\&\approx O\left( T*N*D + T*N^2 \right) \end{aligned}$$

Therefore, the complexity of MISMFO is roughly the same as the regular MFO method in^65^. The space complexity ($$S_{MISMFO}$$) of the proposed MISMFO algorithm is the maximum amount of space used at any time which is considered during its sorting process in each iteration, hence we have $$S_{MISMFO} = O\left( 2*N*D \right)$$, and both MFO and the proposed MISMFO have the same time and space complexity.

## Experiment

In this section, the proposed MISMFO algorithm and other comparative algorithms are evaluated on two test suites. The first test suite is the 15 classical benchmark functions selected from^84^, and the second test suite is the standard IEEE CEC 2020 test set.

To analyze MISMFO, three groups of comparative tests are carried out to evaluate the performance of MISMFO algorithm. Firstly, the convergence and scalability of the MISMFO, along with the basic MFO^65^ and its variants (IMFO^79^, CMFO^70^, WCMFO^69^), are analyzed based on the classical test functions. At the same time, the other 9 meta-heuristic algorithms such as WSO^85^, SCA^86^, DA^87^, GOA^88^, GA^89^, DE^44^, SHADE^90^, LSHADE^91^, COLSHADE^92^ are compared. Secondly, the MISMFO is tested based on CEC 2020 test set and compared with other 13 algorithms. Thirdly, an ablation experiment is conducted on the proposed MISMFO to determine the contribution of each component to its overall performance, followed by a diversity analysis to determine how effectively MISMFO preserves population diversity during the optimization process. Furthermore, a sensitivity analysis is undertaken to examine the robustness of MISMFO in terms of its parameter values, specifically focusing on $\mu$, $\delta _1$ and $\delta _2$. The experiment results indicate that the MISMFO model operates at its best when $\mu = 4$, $\delta _1 = 0.7$ and $\delta _2 = 0.8$. Therefore, in this paper, the values of $\mu$, $\delta _1$ and $\delta _2$ were set to 4, 0.7 and 0.8, respectively. The basic parameters of the algorithms are shown in Table 1. Each algorithm is run independently 30 times for each function, with population size of 30 and maximum number of iterations of 300. All simulation experiments were conducted on Windows 11 Home Edition with a system configuration of AMD Ryzen 7-7840H with Radeon 780M Graphics 3.80GHz. RAM 16.00G, and the language is MATLAB_ R2023a.

**Table 1:** Parameter settings for different algorithms.

Algorithm	Parameter settings
MFO	$b = 1$
IMFO	$\alpha = 0.5$, $\beta = 0.6$, $cr_{max} = 0.65$, $cr_{min} = 0.55$, $sigma_{max} = 0.15$, $sigma_{min} = 0.1$, $R = rand$
WCMFO	$w_{max} = 0.8$, $w_{min} = 0.3$
CMFO	$\mu = 4$, $k =5$
WSO	$f_{max} = 0.75$, $f_{max} = 0.07$, $tau = 4.11$, $p_{min}=0.5$, $p_{max}=1.5$, $a_0=6.25$, $a_1 = 100$, $a_2 = 0.0005$
SCA	$a = 2$
DA	$\beta = 1.5$
GOA	$c_{max}= 1$, $c_{min} = 0.00004$
GA	$pc=0.8, pm=0.05$
DE	$CR = 0.1$, $F = 0.4$
SHADE	$CR = 0.1$, $F = 0.4$, $H = 3$
LSHADE	$CR = 0.1$, $F = 0.4$, $H = 3$
COLSHADE	$CR = 0.1$, $F = 0.4$, $H = 3$
MISMFO	$Rd=0.4$, $\delta _1 = 0.7$, $\delta _2 = 0.8$, $\mu = 4$

### Benchmark function

**Figure 4:**
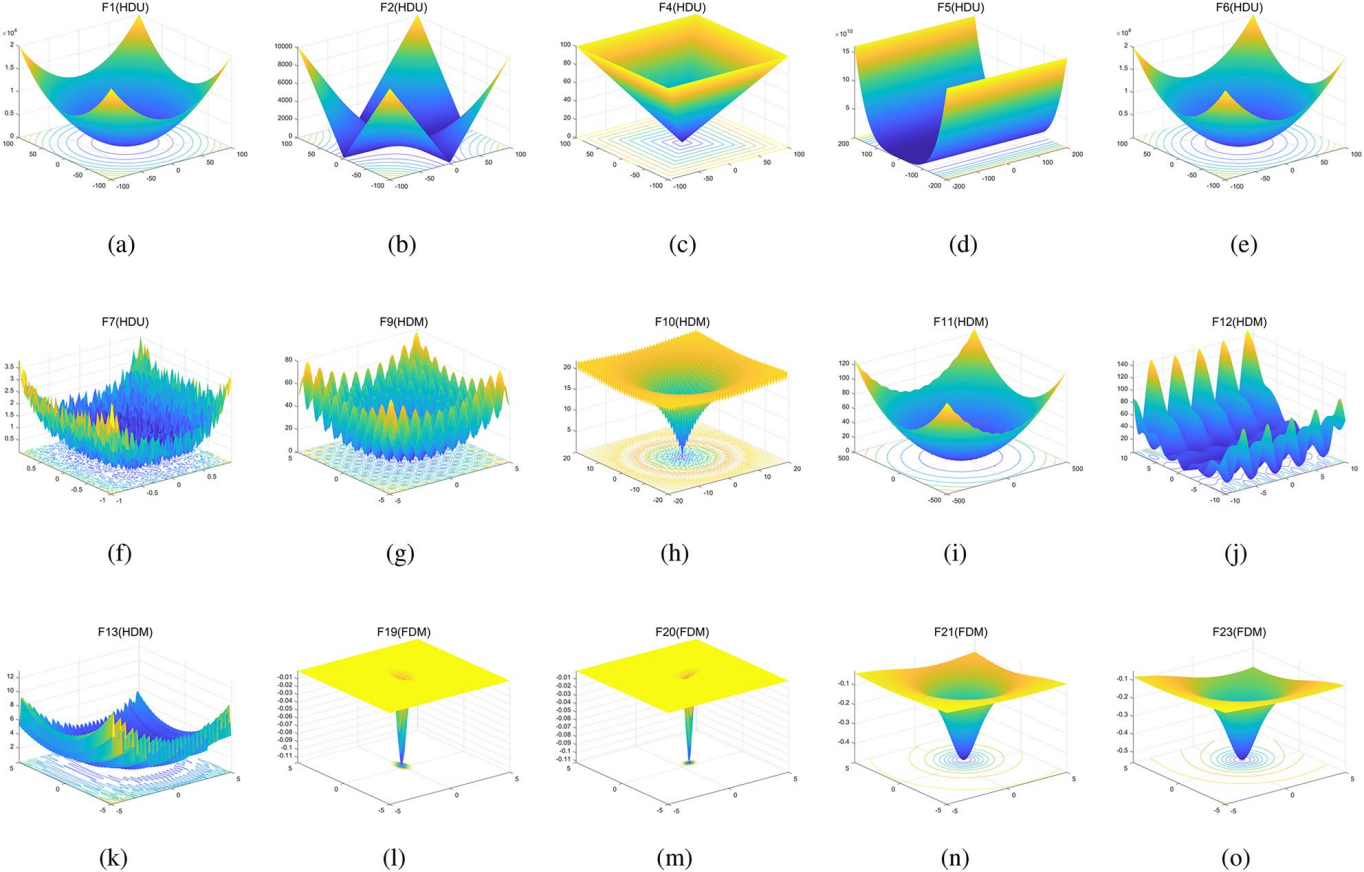
3D graphs of fifteen classical benchmark functions.

In this subsection, the detailed descriptions are provided for both the fifteen classical benchmark functions and the standard CEC 2020 test set, which are utilized to evaluate the algorithms. The 15 classical benchmark problems are divided into three groups: six high-dimensional unimodal functions (F1, F2, F4, F5, F6 F7) to evaluate the local search capability of the algorithms, five high-dimension multimodal functions (F9, F10, F11, F12, F13) to evaluate the global search capability of the algorithms, and four fixed-dimension multimodal functions (F19, F20, F21, F23) to evaluate the convergence performance of the algorithms. The standard CEC 2020 test can be divided into four groups: (1) Uni-modal problems (f1); (2) Multi-modal problems (f2–f4); (3) Hybrid problems (f5–f7); (4) Composition problems (f9–f10). The basic information of the fifteen classical functions and their 3D graphs are shown in Table 2  and Fig. 4, while the summary of the IEEE CEC2020 test functions is shown in Table 3.

**Table 2:** Basic information about the fifteen classical benchmark functions.

Function	Dim	Range	$f_{min}$	Type
$f_1\left( x \right) =\sum _{i=1}^n{x_{i}^{2}}$	[30, 50, 100, 500]	$[-100, 100]$	0	HDU
$f_2\left( x \right) =\sum _{i=1}^n{\left| x_i \right| }+\prod _{i=1}^n{\left| x_i \right| }$	[30, 50, 100, 500]	$[-100, 100]$	0	HDU
$f_4\left( x \right) =\max _i\left\{ \left| x_i \right| ,1\le i\le n \right\}$	[30, 50, 100, 500]	$[-100, 100]$	0	HDU
$f_5\left( x \right) =\sum _{i=1}^{n-1}{\left[ 100\left( x_{i+1}-x_{i}^{2} \right) ^2+\left( x_i-1 \right) ^2 \right] }$	[30, 50, 100, 500]	$[-30, 30]$	0	HDU
$f_6\left( x \right) =\sum _{i=1}^n{\left| x_i+0.5 \right| ^2}$	[30, 50, 100, 500]	$[-100, 100]$	0	HDU
$f_7\left( x \right) =\sum _{i=1}^n{ix^4+random\left[ 0,1 \right) }$	[30, 50, 100, 500]	$[-1.28, 1.28]$	0	HDU
$f_9\left( x \right) =\sum _{i=1}^n{\left[ x_{i}^{2}-10\cos \left( 2\pi x_i \right) +10 \right] }$	[30, 50, 100, 500]	$[-5.12, 5.12]$	0	HDM
$f_{10}\left( x \right) =-20\exp \left( -0.2\sqrt{\left( \frac{1}{n}\sum _{i=1}^n{x_{i}^{2}} \right) } \right) -\exp \left( \frac{1}{n}\sum _{i=1}^n{\cos \left( 2\pi x_i \right) } \right) +20+e$	[30, 50, 100, 500]	$[-32, 32]$	0	HDM
$f_{11}\left( x \right) =\frac{1}{4000}\sum _{i=1}^n{x_{i}^{2}}-\prod _{i=1}^n{\cos \left( \frac{x_i}{\sqrt{i}} \right) }+1$	[30, 50, 100, 500]	$[-600, 600]$	0	HDM
$$f_{12}\left( x \right) =\frac{\pi }{n}\left\{ 10\sin ^2\left( \pi y \right) +\sum _{i=1}^{n-1}{\left( y_i-1 \right) ^2\left[ 1+10\sin ^2\left( \pi y_{i+1} \right) \right] }+\left( y_n-1 \right) ^2 \right\}$$				
$y_i=1+\left( \frac{x_i+1}{4} \right)$	[30, 50, 100, 500]	$[-50, 50]$	0	HDM
$$u\left( x_i,a,k,m \right) ={\left\{ \begin{array}{ll} k\left( x_i-a \right) ^m, x_i>a\\ 0, -a<x_i<a\\ k\left( -x_i-a \right) ^m, x_i<-a\\ \end{array}\right. }$$				
$$f_{13}\left( x \right) =0.1\left\{ \sin ^2\left( 3\pi x_1 \right) +\sum _{i=1}^{n-1}{\begin{array}{c} \left( x_i-1 \right) ^2\left[ 1+\sin ^2\left( 3\pi x_{i+1} \right) \right] \\ +\left( x_n-1 \right) ^2\left[ 1+\sin ^2\left( 2\pi x_n \right) \right] \\ \end{array}} \right\}$$	[30, 50, 100, 500]	$[-50, 50]$	0	HDM
$f_{19}\left( x \right) =-\sum _{i=1}^4{C_i\exp \left( -\sum _{j=1}^3{a_{ij}\left( x_j-P_{ij} \right) ^2} \right) }$	3	[0, 1]	− 3.86278	FDM
$f_{20}\left( x \right) =-\sum _{i=1}^4{C_i\exp \left( -\sum _{j=1}^6{a_{ij}\left( x_j-P_{ij} \right) ^2} \right) }$	6	[0, 1]	− 3.32	FDM
$f_{21}\left( x \right) =-\sum _{i=1}^5{\left[ \sum _{j=1}^4{\left( x_j-a_{ij} \right) ^2+c_i} \right] }^{-1}$	4	[0, 10]	− 10.1532	FDM
$f_{23}\left( x \right) =-\sum _{i=1}^{10}{\left[ \sum _{j=1}^4{\left( x_j-a_{ij} \right) ^2+c_i} \right] }^{-1}$	4	[0, 10]	− 10.5364	FDM

**Table 3:** Summary of the IEEE CEC2020 test functions.

Function	Name of the function	Class	Optimum
f1	Shifted and Rotated Bent Cigar Function	Uni-modal	100
f2	Shifted and Rotated Schwefel’s Function	Multi-modal	1100
f3	Shifted and Rotated Lunacek Bi-Rastrigin Function	Multi-modal	700
f4	Expanded Rosenbrock’s plus Griewangk’s Function	Multi-modal	1900
f5	Hybrid Function 1 ($N=3$)	Hybrid	1700
f6	Hybrid Function 2 ($N=4$)	Hybrid	1600
f7	Hybrid Function 3 ($N=5$)	Hybrid	2100
f8	Composition Function 1 ($N=3$)	Composition	2200
f9	Composition Function 1 ($N=4$)	Composition	2400
f10	Composition Function 1 ($N=5$)	Composition	2500

### Scalability test

In this subsection, based on the benchmark functions (F1, F2, F4, F5, F6, F7, F9, F10, F11, F12, F13), the convergence and scalability of the MISMFO algorithm are analyzed. The MISMFO, the basic MFO, and its variants IMFO, CMFO, and WCMFO are tested across three different dimensions (30, 100, and 500), while all other experimental conditions remain constant. The scalability test evaluates the performance of the algorithm when the problem dimensions and the overall proportion change. The detailed experimental data of the scalability test are shown in Tables 4, 5 and 6, where Avg denotes the mean of 30 independent experiments and Std denotes the standard deviation of 30 independent experiments.

**Figure 5:**
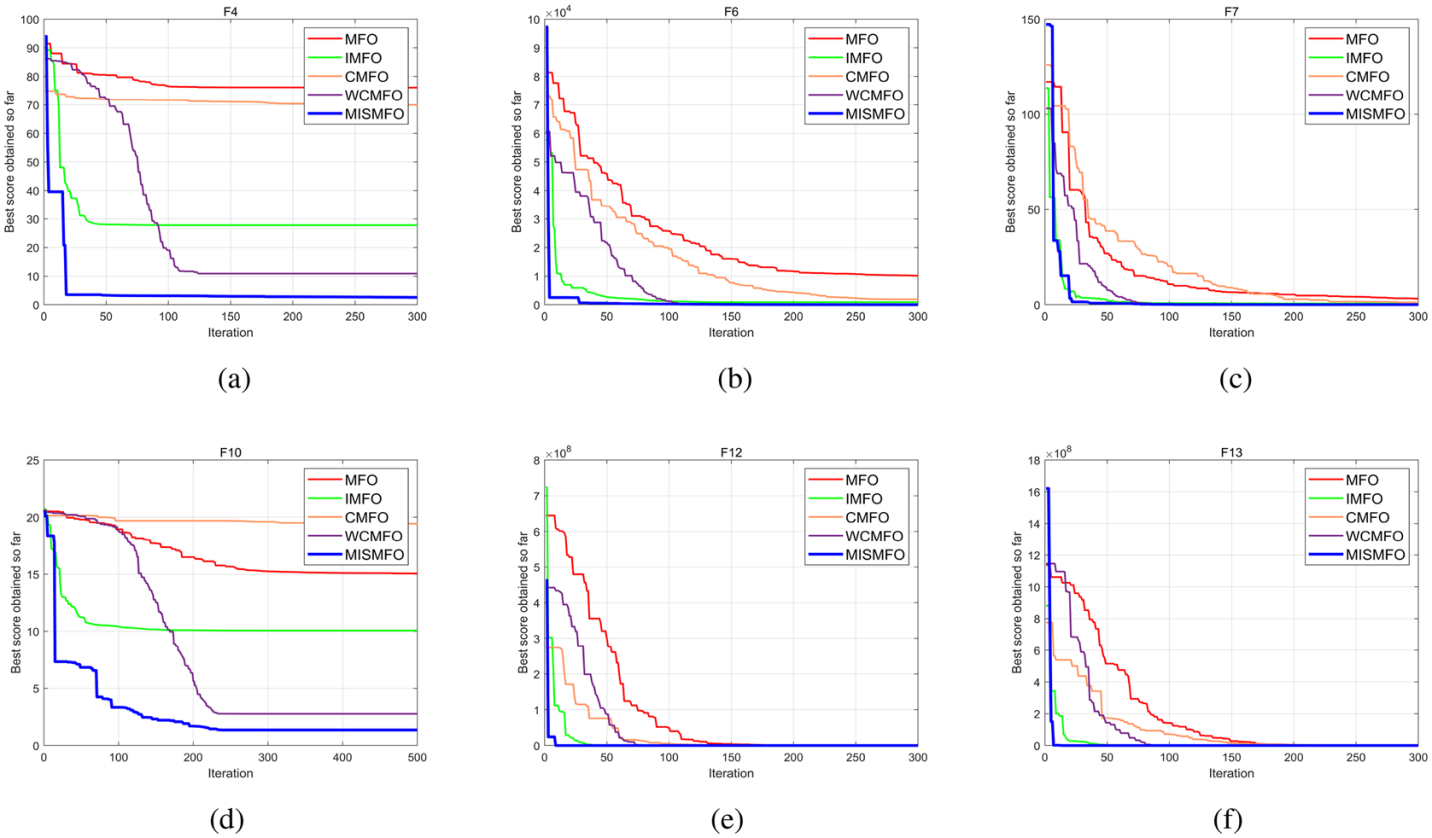
Convergence curves on six functions in dimension 30.

**Figure 6:**
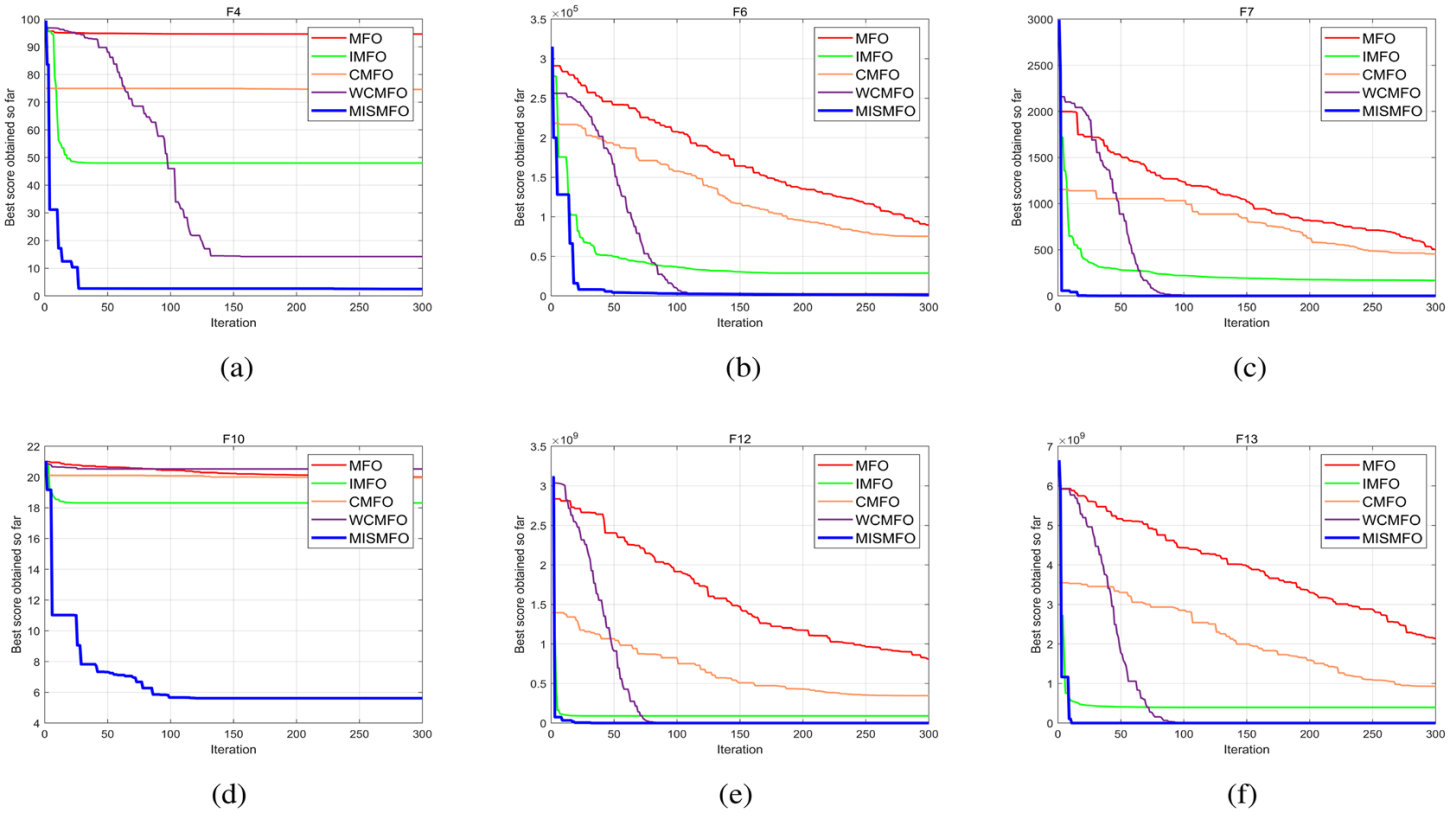
Convergence curves on six functions in dimension 100.

**Figure 7:**
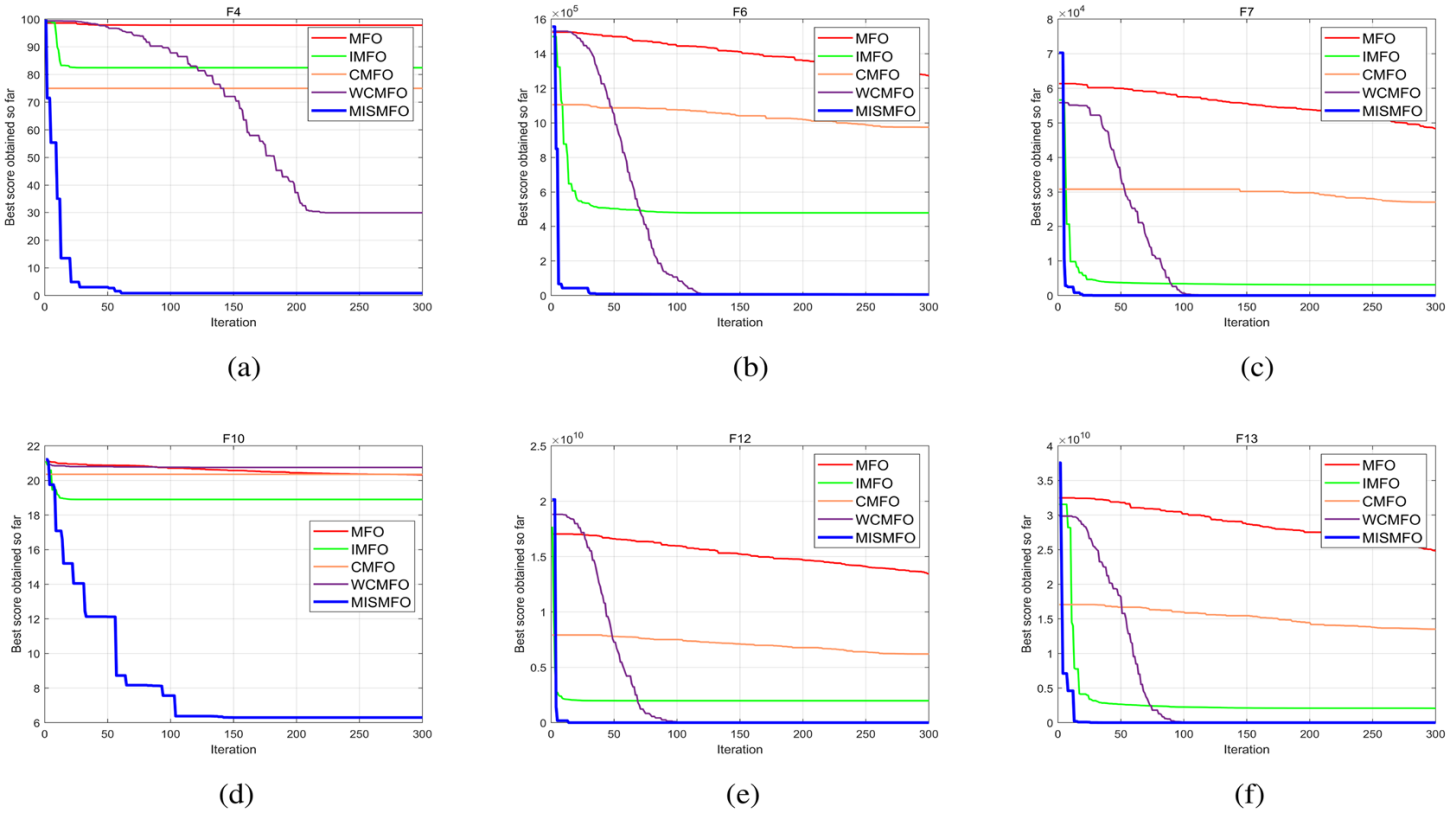
Convergence curves on six functions in dimension 500.

As the dimension of the search space expands, both the Uni-modal benchmark functions and the Multi-modal functions present increasing challenges. WCMFO is better than MISMFO at a dimension of 30 for F7, 100 for F1, and 500 for F2 and F11. However, MISMFO outperforms MFO, IMFO, WCMFO, and CMFO in other dimensional ranges on many problems. In addition, Figs. 5, 6 and 7  show the partial convergence curves of MISMFO, MFO, IMFO, WCMFO and CMFO in different dimensions. These figures clearly show that the convergence speed of the MISMFO algorithm is significantly faster than that of the other four algorithms compared in the scalability test.

MISMFO is capable of finding competitive solutions in both low-dimensional and high-dimensional problems. This may be attributed to the introduction of the Logistic chaos mapping and the flame number phased reduction mechanism, which significantly increase population diversity. Furthermore, the implementation of flame mutation techniques and adaptive position update mechanism enables MISMFO to escape local optimum and more likely achieve a better solution. Taken together, it can be concluded that MISMFO outperforms the basic MFO in different ranges of dimension.

**Table 4:** The scalability test results of five algorithms with 30d.

Dim	Function	Metric	MISMFO	IMFO	WCMFO	CMFO	MFO
Dim=30	F1	Avg	**1.1291E+00**	1.7593E+03	3.8565E+01	6.9401E+03	2.2455E+03
		Std	**1.3913E+00**	1.2295E+03	3.6536E+01	7.8510E+03	4.8171E+03
	F2	Avg	**4.8551E**−**01**	2.0883E+01	1.7788E+00	3.0520E+01	3.0927E+01
		Std	**2.7342E**−**01**	1.3239E+01	1.0081E+00	6.7056E+00	1.7060E+01
	F4	Avg	**2.4064E+00**	3.2466E+01	4.9284E+00	7.7385E+01	7.2919E+01
		Std	**1.5842E+00**	6.2399E+00	2.3445E+00	9.8246E+00	7.5696E+00
	F5	Avg	**1.2161E+03**	8.5194E+07	4.9282E+04	2.6881E+08	3.3934E+08
		Std	**1.7887E+03**	6.4778E+07	7.6960E+04	2.8049E+08	1.8200E+09
	F6	Avg	**7.5333E+00**	2.6919E+03	3.5267E+01	2.9451E+03	3.0481E+03
		Std	**1.5675E+01**	1.4277E+03	3.2092E+01	4.1596E+03	4.3837E+03
	F7	Avg	5.0242E−02	9.8131E−01	**4.3707E**−**02**	1.8313E+00	1.8859E+00
		Std	3.4740E−02	6.0176E−01	**2.0282E**−**02**	9.9565E−01	2.8923E+00
	F9	Avg	**2.9469E+01**	1.2229E+02	1.7371E+02	2.3012E+02	1.7429E+02
		Std	**1.9659E+01**	3.5973E+01	3.4870E+01	3.9347E+01	3.4394E+01
	F10	Avg	**2.0994E+00**	1.3353E+01	1.3256E+01	1.9835E+01	1.4882E+01
		Std	1.0983E+00	3.7094E+00	8.6901E+00	**7.0362E−01**	5.6547E+00
	F11	Avg	**9.5568E**−**01**	1.8401E+01	1.3471E+00	2.3146E+01	2.6649E+01
		Std	**1.3238E**−**01**	7.9771E+00	3.2882E−01	1.7978E+01	3.9996E+01
	F12	Avg	**2.7215E**−**01**	2.5615E+04	1.8153E+00	8.3965E+05	8.7829E+06
		Std	**7.2108E**−**01**	4.9198E+04	1.1254E+00	9.2955E+05	4.6704E+07
	F13	Avg	**1.5302E+00**	7.5243E+05	5.2151E+00	3.2857E+07	7.7353E+05
		Std	2.9086E+00	9.3942E+05	**2.2963E+00**	1.0399E+08	1.6712E+06

Optimal values are in bold.

**Table 5:** The scalability test results of five algorithms with 100d.

Dim	Function	Metric	MISMFO	IMFO	WCMFO	CMFO	MFO
Dim=100	F1	Avg	1.5942E+03	6.3130E+04	**6.7958E+02**	8.3845E+04	1.0084E+05
		Std	8.7698E+02	2.3182E+04	**5.0518E+02**	1.7475E+04	1.3120E+04
	F2	Avg	**2.9667E+01**	2.1629E+02	3.2315E+01	2.6218E+02	3.2032E+02
		Std	**2.7861E+00**	4.0441E+01	1.1176E+01	5.6526E+01	3.6565E+01
	F4	Avg	**1.8758E+00**	4.9864E+01	1.3376E+01	7.4272E+01	9.3429E+01
		Std	1.3542E+00	8.8443E+00	5.0166E+00	**6.1183E**−**01**	1.4343E+00
	F5	Avg	**6.8313E+06**	9.6488E+09	7.3061E+06	2.2592E+10	3.5214E+10
		Std	1.1320E+07	5.8184E+09	**8.7342E+06**	4.8033E+09	8.7387E+09
	F6	Avg	**1.0558E+03**	6.1856E+04	1.1865E+03	8.8123E+04	9.8589E+04
		Std	7.4727E+02	1.8070E+04	**7.4062E+02**	1.4893E+04	1.5472E+04
	F7	Avg	**3.0997E**−**01**	1.0269E+02	4.4635E−01	4.1582E+02	4.2392E+02
		Std	**3.9571E**−**01**	5.3898E+01	4.9761E−01	1.0919E+02	9.1882E+01
	F9	Avg	**2.2666E+02**	8.5569E+02	7.2172E+02	9.1899E+02	9.6888E+02
		Std	9.7588E+01	9.2144E+01	1.2966E+02	**8.9137E+01**	6.5418E+01
	F10	Avg	**6.6000E+00**	1.8178E+01	2.0538E+01	1.9968E+01	1.9965E+01
		Std	1.8216E+00	1.1282E+00	**4.1700E**−**02**	1.4265E−01	6.6725E−02
	F11	Avg	**1.0117E+01**	5.7208E+02	1.1072E+01	8.0135E+02	9.1435E+02
		Std	**6.9906E+00**	1.3528E+02	7.5328E+00	1.4945E+02	1.0591E+02
	F12	Avg	**1.3501E+00**	6.5325E+07	5.0356E+00	3.0657E+08	5.4858E+08
		Std	**1.6555E+00**	5.3390E+07	3.3654E+00	9.0856E+07	2.3250E+08
	F13	Avg	**1.4231E+03**	2.2323E+08	2.1799E+03	7.0814E+08	1.1598E+09
		Std	**6.9540E+03**	9.2197E+07	8.5206E+03	2.1562E+08	2.9240E+08

Optimal values are in bold.

**Table 6:** The scalability test results of five algorithms with 500d.

Dim	Function	Metric	MISMFO	IMFO	WCMFO	CMFO	MFO
Dim=500	F1	Avg	**1.4100E+04**	3.4822E+05	1.6013E+04	9.1291E+05	1.2707E+06
		Std	**8.3330E+03**	8.5681E+04	8.9730E+03	4.5344E+04	3.5256E+04
	F2	Avg	2.9351E+02	1.8225E+75	**1.8592E+02**	7.6066E+20	3.8861E+181
		Std	**2.7631E+01**	9.9822E+75	6.7784E+01	4.1655E+21	INF
	F4	Avg	**1.2124E+00**	8.9053E+01	4.0533E+01	7.4997E+01	9.8945E+01
		Std	8.5823E−01	6.3424E+00	1.0873E+01	**6.3435E**−**03**	2.8321E−01
	F5	Avg	**1.1678E+08**	1.1892E+11	5.0015E+08	3.6247E+11	7.0067E+11
		Std	**2.0363E+08**	5.8788E+10	8.2782E+08	2.2479E+10	3.0713E+10
	F6	Avg	**1.2584E+04**	3.9642E+05	1.4172E+04	9.2179E+05	1.2747E+06
		Std	1.0849E+04	1.1087E+05	**9.1159E+03**	5.0465E+04	3.3745E+04
	F7	Avg	**1.0864E+01**	6.2773E+03	2.5069E+01	2.7550E+04	4.4226E+04
		Std	**1.7680E+01**	2.6032E+03	3.3241E+01	2.1337E+03	2.3396E+03
	F9	Avg	**2.4325E+03**	5.0377E+03	2.6151E+03	6.0311E+03	7.3460E+03
		Std	1.1237E+03	4.2487E+02	7.4240E+02	2.3724E+02	**1.3844E+02**
	F10	Avg	**8.2984E+00**	1.9616E+01	2.0770E+01	2.0288E+01	2.0446E+01
		Std	2.1943E+00	1.1279E+00	**1.4896E**−**02**	5.9565E−02	1.0798E−01
	F11	Avg	1.6086E+02	3.2593E+03	**1.2745E+02**	8.1756E+03	1.1496E+04
		Std	1.0777E+02	7.1208E+02	**6.2159E+01**	3.8314E+02	3.2189E+02
	F12	Avg	**7.0643E+00**	1.2073E+09	2.6392E+05	5.5619E+09	1.3629E+10
		Std	**8.9274E+00**	7.9136E+08	7.8258E+05	4.5326E+08	5.0171E+08
	F13	Avg	**3.0110E+05**	3.1786E+09	2.4728E+06	1.1942E+10	2.5226E+10
		Std	**1.3535E+06**	1.9785E+09	5.7215E+06	7.4326E+08	1.2242E+09

Optimal values are in bold.

### Comparisons on classical benchmark functions

In this subsection, the performance of MISMFO is compared with the basic MFO, its variants IMFO, WCMFO, CMFO, and nine other meta-heuristic algorithms on fifteen benchmark functions. Among these, GA and DE are established as classical algorithms, while WSO, SCA, DA, GOA, DSHADE, LSHADE, and COLSHADE are recognized as novel algorithms proposed in recent years. Additionally, to accurately evaluate the performance differences between these algorithms, both the Friedman test^93^ and the Bonferroni–Dunn test^94^ are conducted on the experimental data from the fifteen benchmark tests.

**Figure 8:**
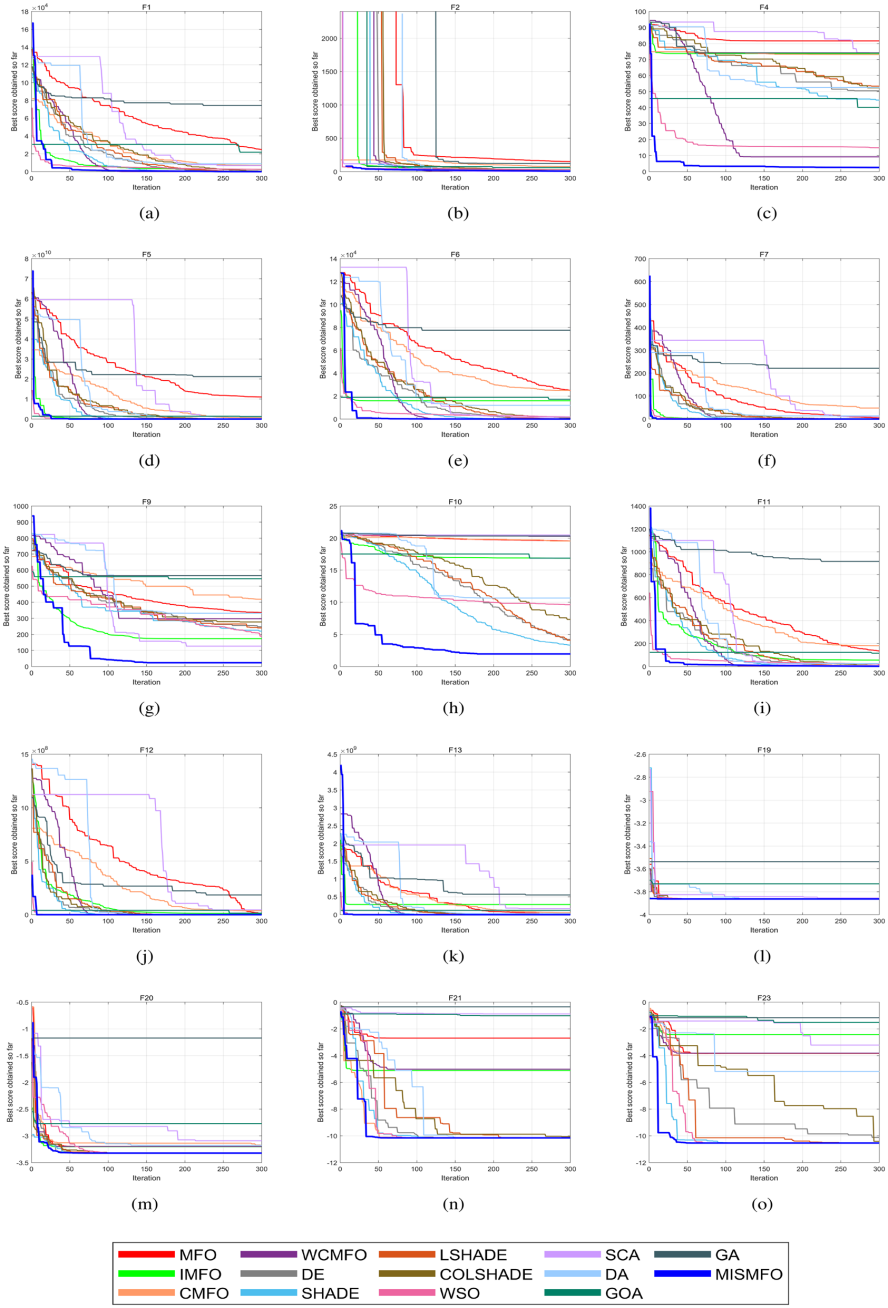
Convergence curves on six functions in dimension 500.

Table 7 shows the results of MISMFO and thirteen other optimization algorithms, each independently executed 30 times on 15 benchmark test functions. The dimensionality is set to 50, with other experimental conditions remaining constant. “Avg” denotes the mean of the test results, while “Std” denotes the standard deviation of the test results. The experimental data shows that MISMFO achieved the highest accuracy on 11 out of 15 test functions. For high-dimensional unimodal functions, MISMFO exhibited superior performance on F4, F5, and F7. Although it did not secure the highest accuracy on F1, F2, and F6, its performance was notably competitive relative to the other 12 algorithms, which indicates that MISMFO has better local search capabilities. For high-dimension multimodal functions F9–F13, MISMFO consistently achieved optimal precision, and its std values demonstrated robust competitiveness, indicating its ability to effectively jump out of the local optima and its superior global search abilities. For the fixed-dimension multimodal functions, MISMFO successfully located the global optima on F19, F21, and F23, and exhibited the smallest std values. Although it did not find the optimal solution on F20, its performance still surpassed most other algorithms, particularly MFO and its variants. This demonstrates that MISMFO possesses considerable stability and convergence capabilities. According to the convergence curves shown in Fig. 8, it can be observed from the figure that MISMFO converges rapidly in the initial stages and continues to search for the optimal values in the later phases.

**Figure 9:**
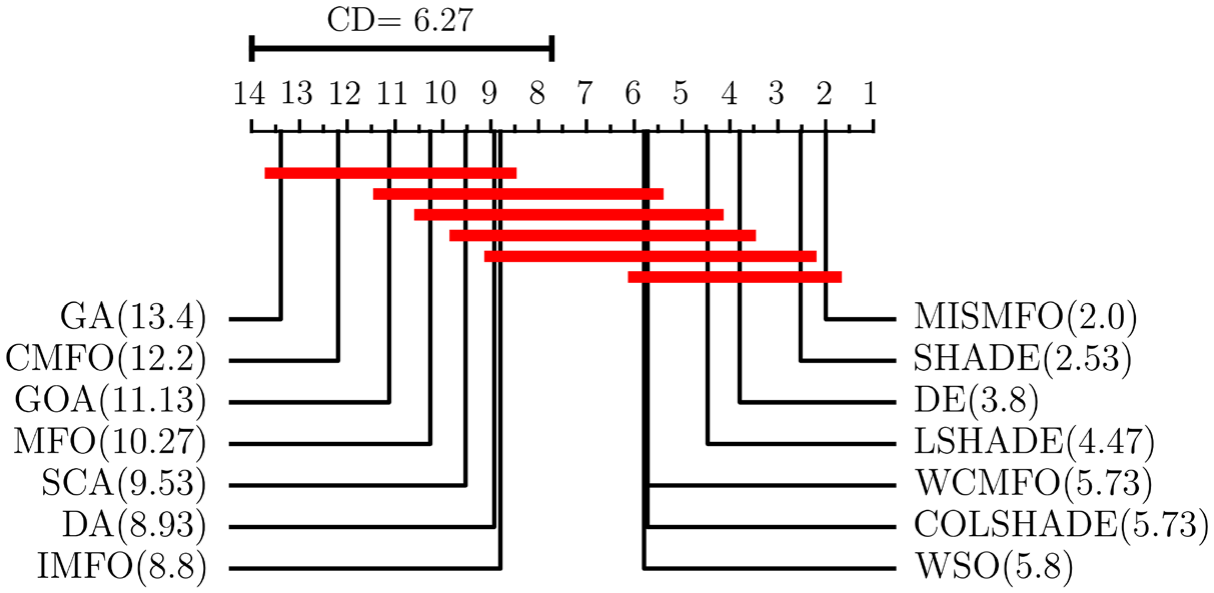
Average rank of different optimization algorithms under Bonferroni–Dunn test on 15 benchmark functions.

Figure 9 presents the Friedman test results of MISMFO compared to other optimization algorithms on fifteen sets of experimental data. The Friedman test at significance level $\alpha = 0.05$, where the original hypothesis indicates that there is no significant difference between this method and the comparison method, and if the original hypothesis is rejected, the Bonferroni Dunn test is used as a $post-hoc$ test to analyze and compare the differences in ranking between the different algorithms. The Critical Difference (CD) is used as a criterion to judge whether there is a significant difference between the different methods, and is calculated as follows:

34 $$\begin{aligned} CD=q_{\alpha }\sqrt{\frac{n\left( n+1 \right) }{6T}}. \end{aligned}$$

where *n* is the number of methods involved in the comparison and *T* is the number of data sets. The significance level of $\alpha = 0.05$ and $n=9$ corresponds to $q_\alpha = 4.1047$, which gives $CD = 6.27(n = 14, T = 15)$. Friedman test is used to assess whether there is a significant difference in the performance of all algorithms on a set of test data. The average rank of MISMFO is 2.0, while the average rank of MFO is 10.27, which indicates a significant improvement in the performance of MISMFO compared to the basic MFO. Among the fourteen algorithms tested, MISMFO is ranked first, which indicates that MISMFO has better search optimization capability compared to the other compared algorithms.

**Table 7:** Comparison of MISMFO with other algorithms on 15 benchmark function.

	F1		F2		F4		F5		F6	
	Avg	Std	Avg	Std	Avg	Std	Avg	Std	Avg	Std
MISMFO	9.6790E+01	6.5604E+01	4.6124E+00	1.0557E+00	**1.6186E+00**	**1.1954E+00**	**1.1601E+05**	**1.6469E+05**	9.1333E+01	6.3575E+01
IMFO	5.4890E+03	2.1653E+03	4.4950E+01	1.8449E+01	4.4741E+01	8.1112E+00	3.3033E+08	1.8666E+08	7.7089E+03	3.0522E+03
WCMFO	2.0740E+02	1.3653E+02	6.2158E+00	2.9231E+00	7.5802E+00	2.9128E+00	8.6198E+05	1.5552E+06	1.5987E+02	1.5158E+02
CMFO	2.7662E+04	9.8976E+03	1.7283E+02	4.8115E+01	8.5498E+01	5.0771E+00	8.4610E+09	6.8211E+09	3.1357E+04	1.6005E+04
MFO	1.5906E+04	9.7737E+03	9.0074E+01	2.4938E+01	8.4128E+01	3.8652E+00	2.2266E+09	3.6791E+09	1.8707E+04	9.2413E+03
DE	1.5548E+02	**3.1299E+01**	4.7136E+00	**4.5998E**−**01**	5.1849E+01	2.5856E+00	1.2619E+06	4.0946E+05	1.6553E+02	**2.2836E+01**
SHADE	**6.6646E+01**	3.7662E+01	2.5170E+00	5.4793E−01	3.8538E+01	2.6500E+00	7.7109E+05	8.9279E+05	**7.4067E+01**	3.9443E+01
LSHADE	1.8440E+02	5.5998E+01	5.7827E+00	8.4501E−01	5.2158E+01	2.8994E+00	1.5986E+06	6.6247E+05	1.9823E+02	5.3429E+01
COLSHADE	9.2714E+02	1.2640E+02	1.3193E+01	1.1673E+00	5.5871E+01	2.4693E+00	2.0191E+07	5.9045E+06	9.3227E+02	1.1502E+02
WSO	2.1853E+03	4.2364E+02	2.2021E+01	3.4134E+00	1.8478E+01	2.3976E+00	2.8773E+07	1.5866E+07	2.2489E+03	6.7633E+02
SCA	2.4320E+03	2.4748E+03	**2.4221E+00**	2.0701E+00	7.6689E+01	5.8519E+00	2.2888E+09	2.4598E+09	2.5872E+03	2.5623E+03
DA	8.1515E+03	3.4285E+03	3.7479E+01	1.3286E+01	4.8033E+01	8.9357E+00	8.4461E+08	8.1391E+08	9.4613E+03	3.6211E+03
GOA	2.0473E+04	9.0260E+03	1.5065E+06	8.2259E+06	4.5546E+01	8.2539E+00	2.5774E+09	1.6968E+09	1.9940E+04	6.8375E+03
GA	8.1801E+04	1.0664E+04	1.2431E+02	1.3067E+01	8.4868E+01	5.0237E+00	2.5778E+10	8.5353E+09	8.1358E+04	1.5367E+04

	F7		F9		F10		F11		F12	
	Avg	Std	Avg	Std	Avg	Std	Avg	Std	Avg	Std
MISMFO	**1.1397E**−**01**	8.9466E−02	**6.1944E+01**	3.0588E+01	**2.7917E+00**	1.3540E+00	**1.6530E+00**	2.8244E−01	**7.3575E**−**01**	9.5812E−01
IMFO	3.5635E+00	1.6277E+00	2.8441E+02	3.4450E+01	1.8624E+01	1.0431E+00	8.8951E+01	3.2883E+01	3.5700E+06	8.4039E+06
WCMFO	1.2247E−01	**5.1650E**−**02**	3.5167E+02	7.0600E+01	1.8316E+01	5.3056E+00	3.1355E+00	1.4159E+00	2.7816E+00	1.0691E+00
CMFO	7.1792E+01	5.5073E+01	5.1738E+02	6.1303E+01	1.9743E+01	4.6372E−01	3.0169E+02	9.1761E+01	3.7412E+07	2.2157E+07
MFO	2.9064E+01	2.9317E+01	3.5824E+02	4.4128E+01	1.9510E+01	6.5801E−01	1.2529E+02	6.8673E+01	4.8131E+07	8.9209E+07
DE	7.1164E−01	1.2998E−01	2.3361E+02	1.3676E+01	4.0482E+00	2.5001E−01	2.3649E+00	**2.0633E**−**01**	6.0772E+00	1.3711E+00
SHADE	2.1045E−01	7.7304E−02	2.0590E+02	**1.1269E+01**	2.9568E+00	3.8772E−01	1.7169E+00	3.4877E−01	2.8839E+00	**9.2438E**−**01**
LSHADE	6.0519E−01	1.4599E−01	2.5428E+02	1.4325E+01	4.3370E+00	2.7063E−01	2.7764E+00	5.5131E−01	7.8314E+00	1.4981E+00
COLSHADE	1.0534E+00	2.3717E−01	2.5746E+02	1.4915E+01	6.9244E+00	2.4891E−01	9.1343E+00	1.2054E+00	2.3299E+01	6.1490E+00
WSO	5.9591E−01	2.5679E−01	2.1842E+02	5.6978E+01	8.6036E+00	7.2217E−01	1.8647E+01	5.5795E+00	1.7750E+01	2.7030E+01
SCA	9.3559E+00	1.0090E+01	1.4673E+02	5.7908E+01	1.3781E+01	7.4009E+00	2.2083E+01	2.3250E+01	3.7948E+07	5.0981E+07
DA	5.1352E+00	3.7431E+00	3.4304E+02	5.2535E+01	1.3718E+01	2.2890E+00	7.3036E+01	4.4531E+01	2.3938E+06	3.3422E+06
GOA	1.9783E+00	1.5212E+00	5.1328E+02	3.7401E+01	1.6265E+01	1.3251E+00	2.0877E+02	1.5744E+02	2.1199E+07	4.4182E+07
GA	1.5855E+02	6.7227E+01	6.4431E+02	6.0076E+01	2.0447E+01	**2.3577E**−**01**	1.9839E+01	3.9424E−01	4.1196E+08	1.6928E+08

	F13		F19		F20		F21		F23	
	Avg	Std	Avg	Std	Avg	Std	Avg	Std	Avg	Std
MISMFO	**1.6764E+01**	3.0885E+01	− **3.8628E+00**	**2.7101E**−**15**	− 3.2546E+00	5.9924E−02	− **1.0153E+01**	**1.8067E**−**15**	− **1.0536E+01**	**9.0336E**−**15**
IMFO	6.8967E+07	4.4141E+07	− **3.8628E+00**	7.6164E−05	− 3.2151E+00	7.8740E−02	− 8.0425E+00	2.6635E+00	− 8.1694E+00	3.4782E+00
WCMFO	1.8912E+01	1.4737E+01	− 3.8624E+00	6.6249E−04	− 3.2323E+00	6.1973E−02	− 6.9104E+00	2.8493E+00	− 8.0623E+00	2.8510E+00
CMFO	3.0503E+08	3.1668E+08	− 3.8624E+00	1.2562E−03	− 3.2239E+00	4.9501E−02	− 5.5164E+00	2.2909E+00	− 5.2676E+00	3.1409E+00
MFO	8.0300E+07	1.6278E+08	− **3.8628E+00**	**2.7101E**−**15**	− 3.2195E+00	6.2245E−02	− 7.0602E+00	3.4573E+00	− 7.7654E+00	3.5375E+00
DE	3.8747E+01	**1.0273E+01**	− **3.8628E+00**	**2.7101E**−**15**	− **3.3220E+00**	5.6298E−05	− 9.8068E+00	1.1643E+00	− 1.0385E+01	2.7211E−01
SHADE	2.6287E+01	1.7575E+01	− **3.8628E+00**	**2.7101E**−**15**	− 3.3219E+00	2.4971E−04	− 9.3106E+00	1.9157E+00	− 1.0535E+01	2.5505E−03
LSHADE	1.3574E+02	2.6474E+02	− **3.8628E+00**	**2.7101E**−**15**	− **3.3220E+00**	**2.3743E**−**05**	− 9.7262E+00	1.2969E+00	− 1.0326E+01	9.8051E−01
COLSHADE	2.6009E+04	1.6308E+04	− **3.8628E+00**	**2.7101E**−**15**	− **3.3220E+00**	7.7278E−05	− 9.9254E+00	2.6469E−01	− 1.0247E+01	2.2994E−01
WSO	1.9054E+04	2.1875E+04	− **3.8628E+00**	4.0901E−15	− 3.3101E+00	3.6278E−02	− 8.9081E+00	2.8316E+00	− 1.0281E+01	1.3995E+00
SCA	8.5027E+07	7.2590E+07	− 3.8537E+00	3.1481E−03	− 2.9164E+00	3.5992E−01	− 2.3263E+00	1.8226E+00	− 3.3573E+00	1.8049E+00
DA	1.7209E+07	1.6777E+07	− 3.8624E+00	1.0597E−03	− 3.2499E+00	1.0405E−01	− 6.1939E+00	2.5338E+00	− 7.5650E+00	2.8487E+00
GOA	5.0788E+07	5.4897E+07	− 3.7257E+00	2.3639E−01	− 2.9297E+00	2.6123E−01	− 2.4697E+00	1.2516E+00	− 3.0630E+00	1.7559E+00
GA	8.5913E+08	3.6271E+08	− 3.3686E+00	3.2601E−01	− 1.5035E+00	5.9981E−01	− 7.8351E−01	5.1430E−01	− 1.1586E+00	3.8598E−01

Optimal values are in bold.

### Comparisons on CEC 2020 test set

**Figure 10:**
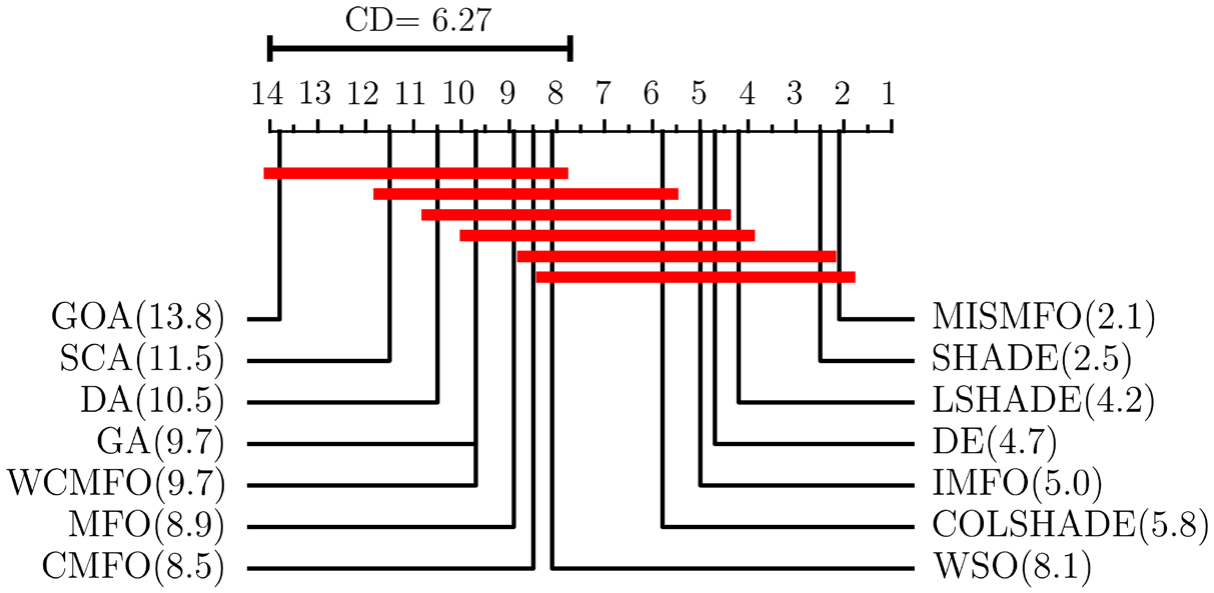
Average rank of different optimization algorithms under Bonferroni–Dunn test on CEC 2020.

To further evaluate the performance of MISMFO, it is analyzed based on CEC 2020 in this section. The MISMFO is compared with the other 13 algorithms (IMFO, WCMFO, CMFO, MFO, DE, SHADE, LSHADE, COLSHADE, WSO, SCA, DA, GOA, GA). Table 8 shows the average and standard deviation of each algorithm running independently 30 times. Figure 10 presents the results of Friedman test for each algorithm on CEC 2020.

According to Table 8, MISMFO achieves the best solutions for f1, f2, f4, f8, f9, and f10 compared to the other 13 algorithms. Although MISMFO does not always find the optimal solution for other problems, its average and std values are highly competitive relative to the other algorithms. Furthermore, the ranking results from 10 test problems clearly show that MISMFO consistently ranks first, outperforming all other algorithms, followed by SHADE, LSHADE, DE, IMFO, COLSHADE, WSO, CMFO, MFO, WCMFO, GA, DA, SCA, and GOA. In summary, it convincingly demonstrates that MISMFO can obtain competitive solutions when solving CEC 2020 problems.

**Table 8:** Comparison of MISMFO with other algorithms on CEC 2020.

	f1		f2		f3		f4		f5	
	Avg	Std	Avg	Std	Avg	Std	Avg	Std	Avg	Std
MISMFO	**1.9755E+04**	**1.9468E+04**	**2.5622E+03**	4.4232E+02	7.8056E+02	1.1651E+01	**1.9060E+03**	1.7882E+00	1.0305E+06	5.1430E+05
IMFO	1.5476E+07	1.9068E+07	3.5142E+03	5.2213E+02	8.5133E+02	6.7765E+01	1.9318E+03	4.2008E+01	1.9474E+05	2.5495E+05
WCMFO	9.1624E+09	1.2601E+09	5.2953E+03	3.0134E+02	9.2299E+02	1.5006E+01	8.1747E+03	2.7338E+03	1.1868E+06	4.3795E+05
CMFO	2.2026E+09	2.1153E+09	3.3994E+03	5.2727E+02	8.9516E+02	1.0243E+02	1.9551E+04	3.0325E+04	3.4262E+06	3.3869E+06
MFO	3.2180E+09	3.6196E+09	3.4072E+03	4.2708E+02	8.6237E+02	1.1045E+02	1.3002E+04	2.5165E+04	2.2667E+06	2.3863E+06
DE	6.8826E+05	3.0390E+05	2.8264E+03	2.2351E+02	7.8121E+02	7.5577E+00	1.9080E+03	1.2598E+00	1.9278E+06	9.8259E+05
SHADE	1.0748E+05	8.6411E+04	2.7732E+03	3.0778E+02	**7.7301E+02**	**6.3528E+00**	1.9061E+03	**7.6661E**−**01**	1.1100E+06	6.0769E+05
LSHADE	5.9304E+05	1.9416E+05	2.9125E+03	**2.1189E+02**	7.8734E+02	8.0172E+00	1.9085E+03	1.0221E+00	1.6516E+06	6.8648E+05
COLSHADE	4.5991E+06	1.1225E+06	2.9324E+03	2.1588E+02	7.9241E+02	9.1179E+00	1.9103E+03	1.4484E+00	1.8761E+06	8.2101E+05
WSO	5.5438E+09	3.5138E+09	5.1862E+03	5.2429E+02	9.0610E+02	5.6938E+01	1.0494E+04	1.3857E+04	**8.7868E+04**	**1.0173E+05**
SCA	1.0196E+10	2.3545E+09	5.5126E+03	2.8454E+02	9.7051E+02	3.7643E+01	7.4452E+03	3.9629E+03	3.2908E+06	1.8791E+06
DA	2.0665E+09	1.0642E+09	4.6838E+03	6.2035E+02	9.1286E+02	6.1859E+01	9.5553E+03	2.3064E+04	3.8347E+06	3.7134E+06
GOA	2.7771E+10	8.5769E+09	6.0050E+03	4.3248E+02	1.2717E+03	1.1904E+02	5.1025E+05	5.9658E+05	1.8153E+07	1.5163E+07
GA	5.8033E+09	3.0294E+09	3.0068E+03	5.7211E+02	9.2218E+02	3.7010E+01	1.8836E+04	2.7527E+04	1.1316E+06	9.7150E+05

	f6		f7		f8		f9		f10	
	Avg	Std	Avg	Std	Avg	Std	Avg	Std	Avg	Std
MISMFO	1.9239E+03	1.8634E+02	4.3797E+05	6.2181E+05	**2.4242E+03**	4.4475E+02	**2.8658E+03**	2.2451E+01	**2.9342E+03**	2.2745E+01
IMFO	2.0030E+03	2.4222E+02	2.4238E+05	6.6909E+05	3.0633E+03	1.2363E+03	2.8840E+03	2.4818E+01	2.9780E+03	4.1634E+01
WCMFO	2.5815E+03	1.5446E+02	3.3811E+05	9.7371E+04	3.2315E+03	1.5148E+02	3.0473E+03	1.7131E+01	3.3716E+03	1.0988E+02
CMFO	2.0728E+03	2.1642E+02	1.0654E+06	9.0441E+05	5.0988E+03	8.1472E+02	2.8796E+03	1.9531E+01	3.0308E+03	1.0081E+02
MFO	2.1626E+03	1.8266E+02	1.2678E+06	1.5955E+06	4.5670E+03	1.2136E+03	2.9065E+03	2.1676E+01	3.1322E+03	1.6567E+02
DE	**1.6223E+03**	**6.1736E+00**	8.3672E+05	3.7208E+05	2.6816E+03	1.2164E+02	2.9111E+03	**1.3238E+01**	2.9804E+03	2.4010E+01
SHADE	1.6323E+03	1.5990E+01	4.3513E+05	2.8571E+05	2.4446E+03	7.6652E+01	2.8843E+03	3.1862E+01	2.9359E+03	1.9767E+01
LSHADE	1.6329E+03	1.4120E+01	6.2847E+05	3.0978E+05	2.5100E+03	**6.3834E+01**	2.9012E+03	4.5484E+01	2.9544E+03	2.3595E+01
COLSHADE	1.6699E+03	2.4821E+01	7.9474E+05	4.6766E+05	2.7092E+03	1.3255E+02	2.9152E+03	3.3972E+01	2.9903E+03	**1.8077E+01**
WSO	2.1762E+03	2.3499E+02	**1.4592E+04**	**1.9362E+04**	3.0841E+03	4.7214E+02	3.0589E+03	6.3583E+01	3.1909E+03	1.6012E+02
SCA	2.5722E+03	2.1188E+02	1.3256E+06	1.2376E+06	5.2210E+03	1.8572E+03	3.0290E+03	2.7518E+01	3.3654E+03	1.5159E+02
DA	2.6799E+03	3.5023E+02	1.2137E+06	1.2953E+06	4.8784E+03	1.8442E+03	3.1641E+03	1.1749E+02	3.1239E+03	1.2853E+02
GOA	3.2170E+03	4.2007E+02	6.9003E+06	4.2083E+06	6.5517E+03	1.2057E+03	3.0564E+03	7.0038E+01	5.5235E+03	1.4427E+03
GA	2.4320E+03	2.7395E+02	2.7485E+06	3.5177E+06	4.0006E+03	1.1784E+03	2.9494E+03	7.4414E+01	3.2720E+03	2.1072E+02

Optimal values are in bold.

### Ablation study

In this subsection, an ablation study is conducted to evaluate the individual contributions of each component within the proposed MISMFO algorithm. The MISMFO algorithm integrates four key components: Logistic chaos mapping for population initialization (MISMFO I), a mutation-based flame update mechanism (MISMFO II), a flame number phased reduction mechanism (MISMFO III), and an adaptive position update mechanism (MISMFO IV). The evaluation was carried out on six classic benchmark functions (F4, F7, F9, F12, F20, F21). For high-dimensional functions (F4, F7, F9, F12), the configuration was set to 30 dimensions with a maximum of 500 iterations, while fixed-dimensional functions (F20, F21) were subjected to 200 iterations.

**Figure 11:**
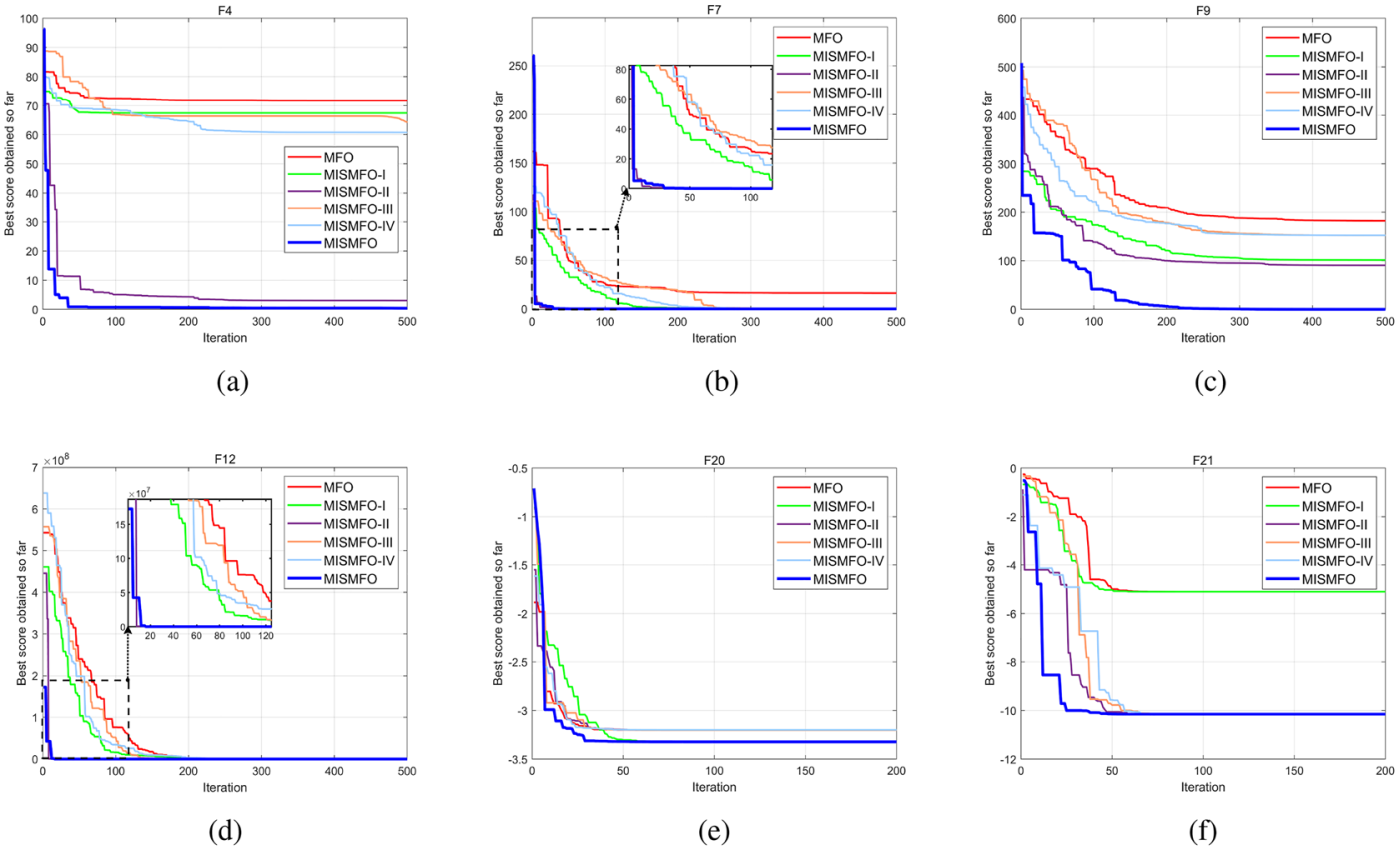
Convergence curves of ablation study for the MISMFO algorithm.

Figure 11 shows the convergence curves of the MISMFO algorithm, its individual components, and the basic MFO across the selected benchmark functions. The results reveal that the mutation-based flame update mechanism (MISMFO II) plays a pivotal role in enhancing the overall performance of the MISMFO algorithm. While the contributions of the other components are beneficial, their impacts are comparatively modest. Overall, each component individually enhances performance relative to the basic MFO, exemplifying the effectiveness of the integrated approach in MISMFO.

The ablation study conclusively underscores the essential role of each component within MISMFO, affirming that their coordinated functionality is crucial for boosting the efficacy and robustness of the algorithm across a variety of testing scenarios.

### Diversity analysis

**Figure 12:**
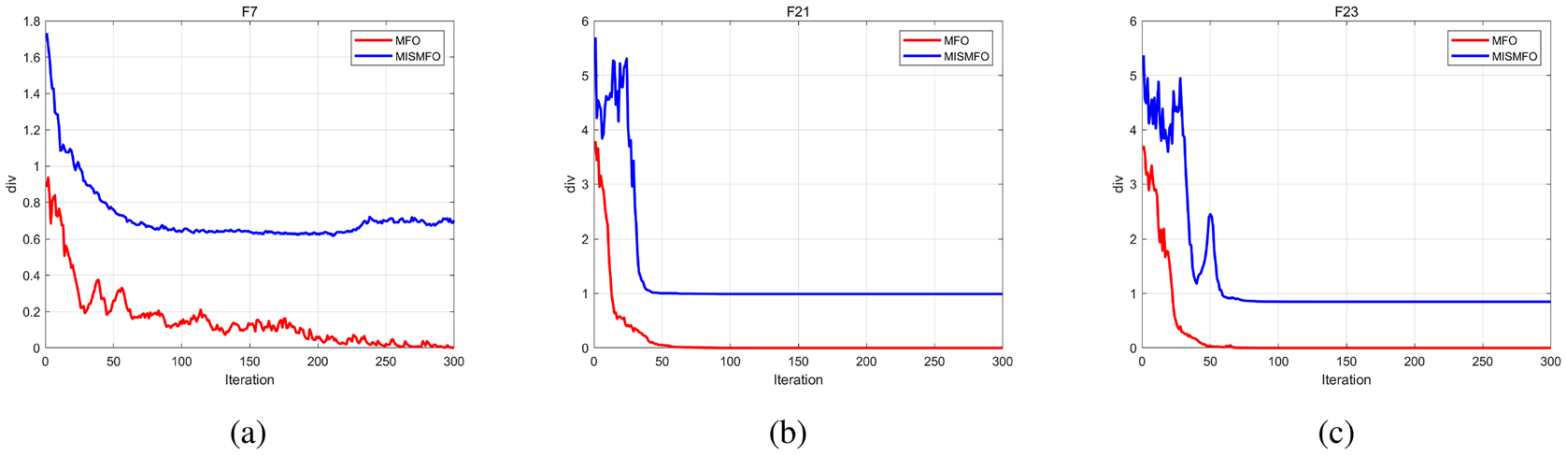
The population diversity monitor of the MISMFO on 3 benchmark functions.

**Figure 13:**
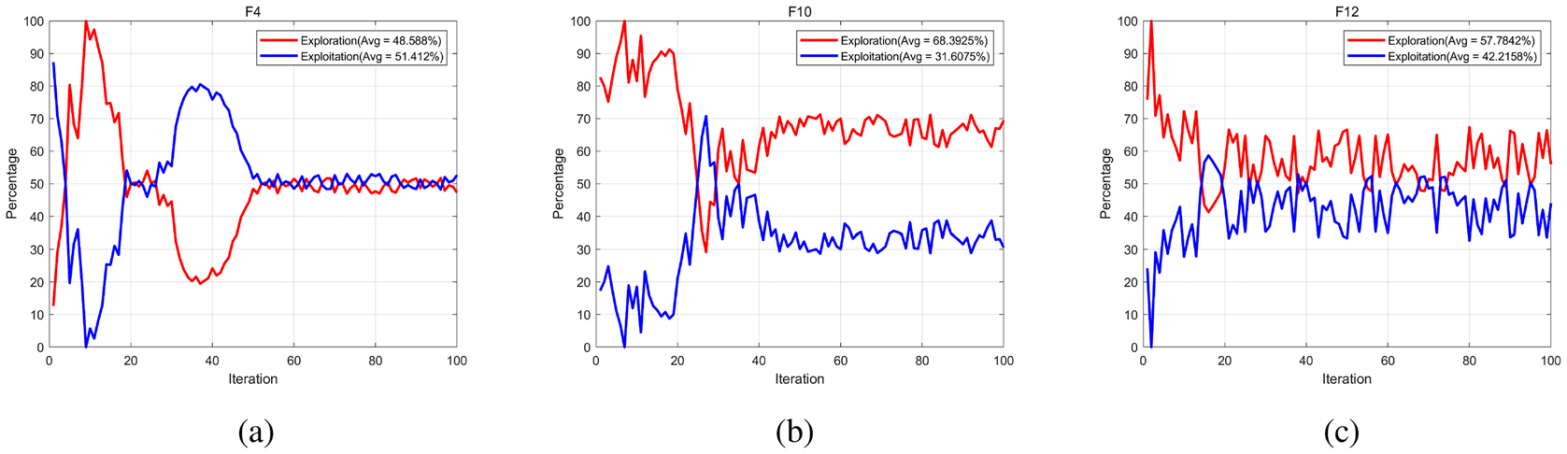
Diversity analysis of MISMFO on 3 benchmark functions.

In this subsection, the diversity and balance of MISMFO were analyzed. Optimization algorithms employ a collective of agents to enhance the exploration of search spaces, thereby accelerating the process of identifying optimal solutions. Typically, agents that discover superior solutions tend to influence the overall direction of the search, promoting convergence. However, this can reduce population diversity, which in turn diminishes the breadth of search areas explored. Conversely, the process of intensification becomes more pronounced as the distances among agents decrease. To assess these dynamics of expanding and contracting distances among search agents, a diversity assessment as described in^59^ is utilized:

35 $$\begin{aligned}{} & {} di{{v}^{j}}=\frac{1}{N}\sum \limits _{i=1}^{N}{\left| median({{x}^{j}})-x_{i}^{j} \right| } \end{aligned}$$

36 $$\begin{aligned}{} & {} div=\frac{1}{d}\sum \limits _{j=1}^{d}{div^{j}} \end{aligned}$$

where *N* and *d* represents number of search agents and design variables respectively, $x_i^j$ is the dimension *j* of the *i*th search agent and $median(x^j)$ is the median of dimension *j* in the whole population, $div^j$ is the diversity in each dimension and mathematically, it is defined as the distance between the *j*th dimension of every search agent and the median of that dimension. The diversity of whole population (*div*) is then calculated by taking average of every $div^j$. Additionally, by employing diversity metrics, it is possible to quantify the proportions of exploration and exploitation in each iteration through the application of the following equations:

37 $$\begin{aligned}{} & {} exploration\%=\left( \frac{div}{di{{v}_{\max }}} \right) \times 100 \end{aligned}$$

38 $$\begin{aligned}{} & {} exploitation\%=\left( \frac{\left| div-di{{v}_{\max }} \right| }{di{{v}_{\max }}} \right) \times 100 \end{aligned}$$

where $div_{max}$ is defined as the maximum diversity value in the whole optimization process and $\left| div-di{{v}_{\max }} \right|$ is the absolute value between *div* and $div_{max}$. The $exploration\%$ is the link between the diversity in each iteration and the maximum diversity obtained. The $exploitation\%$ relates to the exploitation level and it is evaluated as the compliment percentage to $exploration\%$ as the difference between the maximal diversity and the current diversity of an iteration is caused by the concentration of search agents. Figure 12 show the diversity monitoring of MISMFO and MFO on 3 classical benchmark functions (F7, F21, F23). It can be seen that the diversity of MISMFO is consistently maintained at a superior level compared to MFO. During the search process, the diversity value of the MISMFO population is maintained at a high level, which enhances the capability of the MISMFO to escape local optima. According to Fig. 13, the proposed MISMFO algorithm was evaluated on 3 classical benchmark functions (F4, F10, F12), facilitating an analysis of the trade-offs between exploration and exploitation. The X-axis represents the number of iterations, while the Y-axis depicts the percentage of both exploration and exploitation activities. It is observable that the MISMFO algorithm initially exhibits substantial exploration capabilities, which progressively shift towards enhanced exploitation as the iterations advance. Before convergence, the algorithm maintains a balance between exploration and exploitation, with exploitation becoming increasingly dominant over exploration. This demonstrates that MISMFO effectively maintains a balance between exploration and exploitation, thereby swiftly locating optimal solutions to problems.

### Sensitivity analysis

In this subsection, a sensitivity analysis is performed to evaluate the impacts of critical parameters on the performance of the MISMFO algorithm on three classical benchmark functions (F4, F7, F10). The parameters analyzed include the bifurcation parameter $\mu$($\mu \in [0, 4]$), and the phase division factors $\delta _1$ and $\delta _2$ ($0 \le \delta _1 \le \delta _2 \le 1$). The objective of this analysis is to ascertain the robustness of MISMFO to variations in these parameters and to identify settings that optimize performance. The analysis was structured into two distinct parts: firstly, assessing the effect of varying $\mu$ while maintaining constant values for $\delta _1$ and $\delta _2$. Secondly, evaluating the impact of various combinations of $\delta _1$ and $\delta _2$ with a fixed $\mu$. According to Fig. 1, which shows that the Logistic chaotic map stabilizes at zero and exhibits non-chaotic behavior when $\mu$ is within the range of [0, 1], the parameter $\mu$ was consequently adjusted in [1, 4] to enable a more comprehensive analysis.

**Figure 14:**
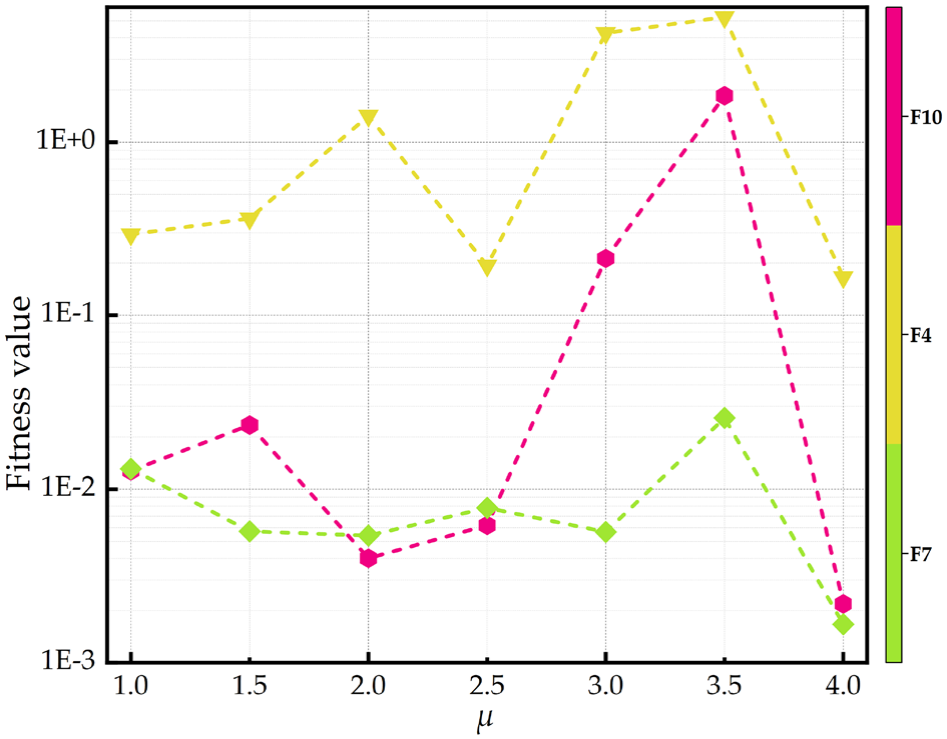
Sensitivity analysis of parameter $\mu$ for the MISMFO.

**Figure 15:**
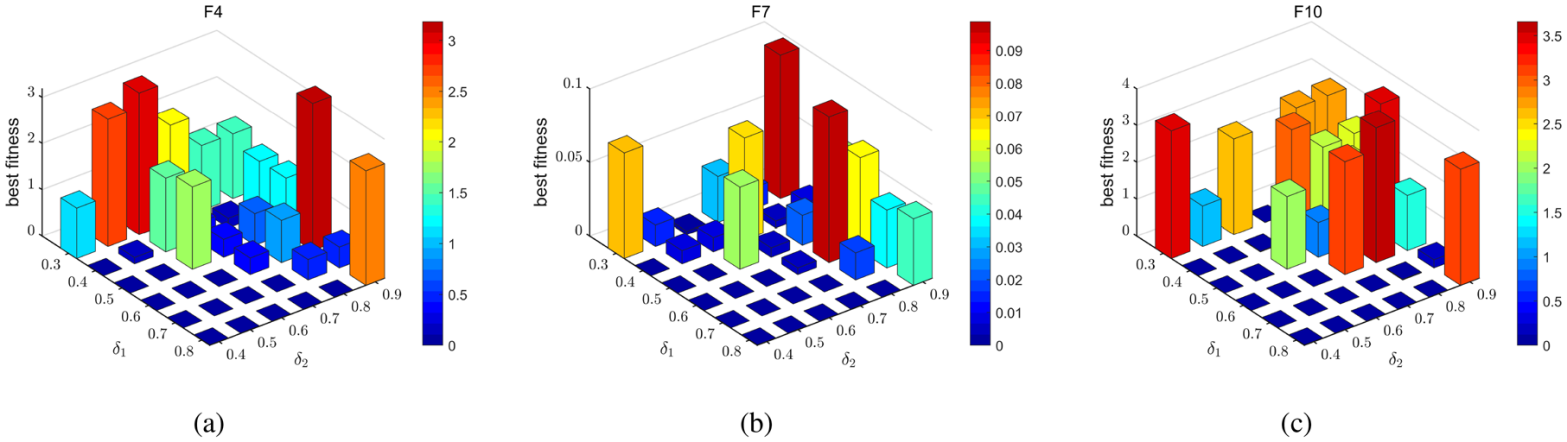
Sensitivity analysis of parameters $\delta _1$ and $\delta _2$ for the MISMFO (since the condition $0 \le \delta _1 \le \delta _2 \le 1$, there are no bars at points outside the condition).

Figures 14 and 15 show the results of sensitivity test. Figure 14  reveals that the accuracy of MISMFO significantly fluctuates with varying values of $\mu$, demonstrating pronounced sensitivity to this parameter. The algorithm achieves optimal performance at $\mu = 4$, indicating that this is the most effective setting for $\mu$ within the evaluated range. Figure 15 illustrates the effects of varying combinations of $\delta _1$ and $\delta _2$ on the performance of the MISMFO algorithm, demonstrating that these parameters significantly influence its accuracy. Extensive experimentation has revealed that the algorithm performs optimally when $\delta _1$ is set to 0.7 and $\delta _2$ to 0.8, which corresponds to the settings where MISMFO achieves its peak performance. This underscores the importance of precise parameter tuning to optimize the effectiveness of the MISMFO algorithm.

## Case study

In this section, the MISMFO-MKSVR model is applied to estimate the effort of the software projects to validate its ability to solve practical problems.

### Description of the data sets

Five publicly available datasets from PROMISE (http://promise.site.uottawa.ca/SERepository) are selected for testing the proposed MISMFO-MKSVR model. These five datasets are COCOMO81, Maxwell, Desharnais, Miyazaki and China. The attributes of these datasets can be broadly classified into two categories, numerical and categorical. Table 9 summarises the main features of the selected datasets, including name, number of cases, number of numerical attributes, number of categorical attributes, and the unit of effort (Table 9).

**Table 9:** Description of the data sets.

Datasets	Cases	nbrNum	nbrCat	Unit Effort
COCOMO81	63	1	16	Person-months
Maxwell	62	2	24	Person-hours
Desharnais	80	10	None	Person-hours
Miyazaki	48	7	None	Person-months
China	499	16	None	Person-hours

### Evaluation criteria

In order to evaluate and compare the accuracy of the proposed model, this paper chooses MMRE, MdMRE^3^, MAE^24^, RMSE and $R^2$^23^ as the evaluation indexes for this experiment, and the formula for each index is as follows:

39 $$\begin{aligned} MMRE= & {} \frac{1}{n}\sum _{i=1}^n{\frac{\left| y_i-\hat{y}_i \right| }{y_i}} \end{aligned}$$

40 $$\begin{aligned} MdMRE= & {} median\left( \frac{\left| y_i-\hat{y}_i \right| }{y_i} \right) \end{aligned}$$

41 $$\begin{aligned} MAE= & {} \frac{1}{n}\sum _{i=1}^n{\left| y_i-\hat{y}_i \right| } \end{aligned}$$

42 $$\begin{aligned} RMSE= & {} \sqrt{\frac{1}{n}\sum _{i=1}^n{(y_i-\hat{y}_i)^2}} \end{aligned}$$

43 $$\begin{aligned} R^2= & {} 1-\frac{\sum _{i=1}^n{(y_i-\hat{y}_i)^2}}{\sum _{i=1}^n{(y_i-\bar{y}_i)^2}} \end{aligned}$$

where $y_i$ is the *i*-th true value, $\hat{y}_i$ is the *i*-th predicted value, *n* is the test sample size and $\bar{y}_i$ is the mean values of *n* test samples.

### Comparisons with other optimization algorithms

In this subsection, the proposed MISMFO-MKSVR model and the MKSVR model optimized by other thirteen optimization algorithms (IMFO, WCMFO, CMFO, MFO, DE, SHADE, LSHADE, COLSHADE, WSO, SCA, DA, GOA, GA) are used to estimate the software effort on five public datasets, then the estimation results are compared by MMRE, MdMRE, $R^2$, RMSE, and MAE. The datasets are divided into training and testing sets, and the sample proportion of the training set for this experiment is 70%. The experimental parameters of each algorithm are set unchanged, and the specific experimental results are shown in Tables 10, 11, 12, 13 and 14.

As can be seen in Tables 10, 11, 12, 13 and 14, the proposed MISMFO-MKSVR model performs best on Desharnais in all five metrics. On the COCOMO81 dataset, the MISMFO-MKSVR model exhibits superior performance on four metrics, among which it significantly outperforms other algorithms on RMSE. While it does not attain the best results on $R^2$, it consistently outperforms the majority of competing algorithms. On the Miyazaki dataset, despite the fact that MISMFO-MKSVR fails to be optimal on MdMRE, it significantly surpasses the performance of most competing algorithms, particularly on RMSE. On the Maxwell dataset, the results demonstrate that the MISMFO-MKSVR achieves the best performance across all four evaluated metrics. While it slightly under-performs compared to SHADE-MKSVR on MAE, it still outstrips the majority of the competing algorithms, particularly the basic MFO and its variants.

On the China dataset, the results analysis reveals that MISMFO-MKSVR achieved the best performance on RMSE. Although it did not attain the optimal results on other metrics, its outcomes were very close to the best values and significantly better than those of most competing algorithms. Moreover, it exhibits significant performance enhancement compared to the basic MFO and its variants.

It is experimentally verified that MISMFO can effectively solve the parameter optimization problem of MKSVR. Meanwhile, when the MISMFO-MKSVR model is used to estimate the software effort on five public datasets, the estimation accuracy and fitting ability of MISMFO-MKSVR are markedly better than those of MKSVR prediction models optimized by the basic MFO and other algorithms. It means that the MISMFO-MKSVR model has strong competitiveness in estimation performance compared with the other 13 models, and can solve the problems of software effort estimation.

**Table 10:** MMRE values on different Software data sets based on different models.

MMRE	COCOMO81	Maxwell	Desharnais	China	Miyazaki
MISMFO-MKSVR	**8.7523E**−**01**	**8.1102E**−**01**	**6.5664E**−**01**	4.0619E−01	**4.5370E**−**01**
IMFO-MKSVR	9.9290E−01	8.4204E−01	7.1385E−01	5.6465E−01	4.9683E−01
WCMFO-MKSVR	8.9441E−01	8.3948E−01	7.8235E−01	5.0611E−01	4.6001E−01
CMFO-MKSVR	8.8861E−01	1.0629E+00	1.2504E+00	5.2592E−01	5.3651E−01
MFO-MKSVR	9.5630E−01	1.2909E+00	1.7086E+00	5.6268E−01	6.5607E−01
DE-MKSVR	2.3076E+00	1.1405E+00	1.0565E+00	1.7893E−01	5.2060E−01
SHADE-MKSVR	2.2084E+00	8.7515E−01	1.1775E+00	1.2818E−01	4.6761E−01
LSHADE-MKSVR	2.7488E+00	1.0095E+00	1.2297E+00	1.3181E−01	7.1150E−01
COLSHADE-MKSVR	3.1529E+00	1.2433E+00	1.2192E+00	**1.2306E**−**01**	6.1783E−01
WSO-MKSVR	1.1307E+00	9.2080E−01	8.0616E−01	9.7096E−01	8.3124E−01
SCA-MKSVR	2.5486E+00	1.1456E+00	3.6788E+00	5.0119E−01	6.9335E−01
DA-MKSVR	1.6305E+00	1.1313E+00	3.0209E+00	7.2153E−01	1.3670E+00
GOA-MKSVR	2.1019E+00	1.3612E+00	1.0676E+01	1.0845E+00	1.8392E+00
GA-MKSVR	1.2876E+00	1.1323E+00	1.1172E+00	9.4071E−01	8.0704E−01

Optimal values are in bold.

**Table 11:** MdMRE values on different Software data sets based on different models.

MdMRE	COCOMO81	Maxwell	Desharnais	China	Miyazaki
MISMFO-MKSVR	**6.8146E**−**01**	**5.5127E**−**01**	**3.8504E**−**01**	3.3681E−01	3.4222E−01
IMFO-MKSVR	7.1557E−01	7.0406E−01	4.3524E−01	4.6531E−01	4.3467E−01
WCMFO-MKSVR	6.9275E−01	5.6911E−01	4.7814E−01	3.8329E−01	**3.2851E**−**01**
CMFO-MKSVR	7.1149E−01	7.2743E−01	5.6871E−01	4.4343E−01	4.6083E−01
MFO-MKSVR	6.8275E−01	7.1724E−01	6.5251E−01	4.5191E−01	5.0780E−01
DE-MKSVR	1.1036E+00	6.4343E−01	5.7977E−01	9.8243E−02	3.5729E−01
SHADE-MKSVR	1.0756E+00	5.9690E−01	6.4878E−01	4.0870E−02	3.4662E−01
LSHADE-MKSVR	1.6588E+00	6.6565E−01	6.3055E−01	7.2395E−02	3.4281E−01
COLSHADE-MKSVR	1.4772E+00	6.0794E−01	5.7549E−01	**3.3131E**−**02**	3.4901E−01
WSO-MKSVR	7.3092E−01	6.6820E−01	5.1597E−01	7.1412E−01	6.2727E−01
SCA-MKSVR	9.6526E−01	7.1422E−01	1.4512E+00	3.7529E−01	4.3897E−01
DA-MKSVR	8.7448E−01	6.7032E−01	1.1915E+00	5.8637E−01	5.6269E−01
GOA-MKSVR	8.9469E−01	6.7118E−01	3.8033E+00	7.2557E−01	6.0581E−01
GA-MKSVR	7.6078E−01	6.6951E−01	6.3569E−01	6.9040E−01	6.4837E−01

Optimal values are in bold.

**Table 12:** $R^2$ values on different Software data sets based on different models.

$R^2$	COCOMO81	Maxwell	Desharnais	China	Miyazaki
MISMFO-MKSVR	5.1655E−01	**6.8107E**−**01**	**6.2088E**−**01**	6.3690E−01	**8.4133E**−**01**
IMFO-MKSVR	4.4694E−01	6.7195E−01	5.5923E−01	5.4214E−01	7.4430E−01
WCMFO-MKSVR	4.6517E−01	6.6606E−01	5.2423E−01	4.7621E−01	7.0089E−01
CMFO-MKSVR	4.5902E−01	6.3494E−01	4.9552E−01	5.0202E−01	7.4410E−01
MFO-MKSVR	4.7448E−01	6.0736E−01	4.6091E−01	4.5394E−01	6.7405E−01
DE-MKSVR	5.6235E−01	6.6964E−01	3.5525E−01	**6.8261E**−**01**	7.4880E−01
SHADE-MKSVR	5.2653E−01	6.7251E−01	3.6357E−01	6.5114E−01	6.8120E−01
LSHADE-MKSVR	5.4157E−01	6.7404E−01	4.2064E−01	5.2752E−01	7.4437E−01
COLSHADE-MKSVR	**5.8163E**−**01**	6.6657E−01	3.6298E−01	5.8593E−01	7.7331E−01
WSO-MKSVR	4.4440E−01	6.4226E−01	5.5152E−01	5.3100E−01	6.5976E−01
SCA-MKSVR	4.2258E−01	6.2445E−01	3.0923E−01	4.6172E−01	6.0949E−01
DA-MKSVR	4.3537E−01	6.4823E−01	4.0913E−01	5.3009E−01	5.6517E−01
GOA-MKSVR	4.8435E−01	6.0843E−01	2.2778E−01	5.1832E−01	6.7935E−01
GA-MKSVR	4.9933E−01	6.6286E−01	4.1852E−01	6.0454E−01	6.4267E−01

Optimal values are in bold.

**Table 13:** RMSE values on different Software data sets based on different models.

RMSE	COCOMO81	Maxwell	Desharnais	China	Miyazaki
MISMFO-MKSVR	**8.9386E**−**02**	**1.0091E**−**01**	**1.1046E**−**01**	**8.2079E**−**02**	**8.1746E**−**02**
IMFO-MKSVR	1.5578E−01	1.2895E−01	1.2345E−01	9.6618E−02	1.0070E−01
WCMFO-MKSVR	1.0334E−01	1.2661E−01	1.2790E−01	9.3233E−02	9.7849E−02
CMFO-MKSVR	1.1673E−01	1.3185E−01	1.5036E−01	9.5298E−02	1.0317E−01
MFO-MKSVR	1.0633E−01	1.3772E−01	1.5234E−01	1.0836E−01	1.5887E−01
DE-MKSVR	1.3985E−01	1.0746E−01	1.7196E−01	8.9263E−02	2.9369E−01
SHADE-MKSVR	1.0941E−01	1.0710E−01	3.2813E−01	8.9591E−02	3.6568E−01
LSHADE-MKSVR	1.2172E+00	1.0850E−01	3.8733E−01	1.1228E−01	3.3062E−01
COLSHADE-MKSVR	9.4375E−01	1.0923E−01	3.5327E−01	9.9188E−02	4.9115E−01
WSO-MKSVR	1.0309E−01	1.3019E−01	1.4997E−01	1.0439E−01	4.0005E−01
SCA-MKSVR	1.3961E−01	1.3118E−01	3.2551E−01	9.7110E−02	1.0426E−01
DA-MKSVR	1.3120E−01	1.3414E−01	3.0346E−01	1.0506E−01	3.0301E−01
GOA-MKSVR	1.3112E−01	1.4239E−01	1.1635E+00	9.9257E−02	1.2674E−01
GA-MKSVR	1.1564E−01	1.3482E−01	1.8726E−01	9.4752E−02	3.8606E−01

Optimal values are in bold.

**Table 14:** MAE values on different Software data sets based on different models.

MAE	COCOMO81	Maxwell	Desharnais	China	Miyazaki
MISMFO-MKSVR	**4.9140E**−**02**	7.3929E−02	**7.6498E**−**02**	3.8111E−02	**3.3847E**−**02**
IMFO-MKSVR	8.0150E−02	7.5931E−02	8.4435E−02	4.7310E−02	3.5151E−02
WCMFO-MKSVR	5.0936E−02	7.4980E−02	8.8518E−02	4.1125E−02	3.8766E−02
CMFO-MKSVR	5.3825E−02	8.6666E−02	1.1672E−01	4.4069E−02	4.1822E−02
MFO-MKSVR	5.2324E−02	9.2527E−02	1.1258E−01	4.8137E−02	5.9479E−02
DE-MKSVR	7.2787E−02	7.4829E−02	1.2019E−01	2.3726E−02	1.0742E−01
SHADE-MKSVR	6.5283E−02	**7.3182E**−**02**	1.4114E−01	2.0557E−02	1.0928E−01
LSHADE-MKSVR	2.0671E−01	7.3863E−02	2.0873E−01	2.7502E−02	1.0818E−01
COLSHADE-MKSVR	2.8563E−01	7.4590E−02	1.3970E−01	**1.8667E**−**02**	1.4562E−01
WSO-MKSVR	5.2391E−02	8.3620E−02	1.0216E−01	5.5508E−02	1.3334E−01
SCA-MKSVR	6.9561E−02	8.6335E−02	3.4071E−01	4.3727E−02	4.2882E−02
DA-MKSVR	6.1431E−02	8.5086E−02	1.7657E−01	5.1475E−02	1.2308E−01
GOA-MKSVR	7.4005E−02	8.9148E−02	8.3969E−01	5.9228E−02	7.1721E−02
GA-MKSVR	5.9846E−02	8.7183E−02	1.1471E−01	4.9796E−02	1.2304E−01

Optimal values are in bold.

### Comparisons with other software effort estimation methods

In this subsection, the performance of the proposed MISMFO-MKSVR model is compared with five established software effort prediction models (SVR-Poly, SVR-Linear, SVR-RBF, Linear Regression, ANN) as delineated in^29^ on five public datasets. The experimental parameters remain unchanged, and the results are presented in Table 15. The results clearly show the superior performance of the MISMFO-MKSVR model. On the Maxwell, Desharnais, and Miyazaki datasets, MISMFO-MKSVR achieves the best performance on all five indicators. On the COCOMO81 dataset, Although MISMFO-MKSVR does not attain the leading performance on $R^2$, it remains exceedingly competitive relative to other methods. On the China dataset, the MISMFO-MKSVR model outperforms on RMSE, MAE and $R^2$, while it slightly under-performs compared to SVR-Poly on MMRE and MdMRE, it is very close to the best values. Moreover, it demonstrates considerable competitiveness relative to other methods. This experiment demonstrates that MKSVR generally outperforms single-kernel SVR across a majority of scenarios. The results support the adoption of MKSVR as a more effective tool for capturing the complexities inherent in software projects, thereby enhancing the accuracy of predictive analytics. The under-performance of ANN in this experiment may be attributed to its parameter settings, such as the number of hidden layers, underscoring the importance of parameter optimization. Overall, the MISMFO-MKSVR model exhibits outstanding suitability and effectiveness in addressing software effort prediction challenges.

**Table 15:** Different metrics values on different Software data sets based on different software effort estimation models.

	COCOMO81	Maxwell	Desharnais	China	Miyazaki
MMRE					
MISMFO-MKSVR	**8.7523E**−**01**	**8.1102E**−**01**	**6.5664E**−**01**	4.0619E−01	**4.5370E**−**01**
SVR-Poly	2.6304E+00	1.1162E+00	8.2436E−01	**2.1018E**−**01**	4.5604E−01
SVR-Linear	1.4719E+00	1.0655E+00	6.8272E−01	4.1757E−01	4.8727E−01
SVR-RBF	8.8098E−01	1.0496E+00	6.6522E−01	4.0963E−01	4.9120E−01
Linear-Regression	4.4938E+00	2.1881E+00	9.4369E−01	6.3410E−01	5.2106E−01
ANN	6.6032E+00	3.1659E+00	2.0221E+00	2.1771E+00	1.0022E+00
MdMRE					
MISMFO-MKSVR	**6.8146E**−**01**	**5.5127E**−**01**	**3.8504E**−**01**	3.3681E−01	**3.4222E**−**01**
SVR-Poly	8.9251E−01	6.0779E−01	4.0169E−01	**1.2387E**−**01**	3.5818E−01
SVR-Linear	7.4033E−01	5.6681E−01	3.9298E−01	2.8729E−01	3.7291E−01
SVR-RBF	6.8842E−01	5.5870E−01	3.8827E−01	2.9552E−01	3.6459E−01
Linear-Regression	2.3948E+00	7.4024E−01	4.4770E−01	3.8373E−01	4.3866E−01
ANN	2.6217E+00	1.2388E+00	7.8579E−01	7.8864E−01	5.6707E−01
$$R^2$$					
MISMFO-MKSVR	5.1655E−01	**6.8107E**−**01**	**6.2088E**−**01**	**6.3690E**−**01**	**8.4133E**−**01**
SVR-Poly	**6.0438E**−**01**	6.7597E−01	4.5622E−01	6.2872E−01	8.0113E−01
SVR-Linear	4.8912E−01	6.7143E−01	5.6245E−01	6.0441E−01	7.2244E−01
SVR-RBF	2.1679E−01	5.0740E−01	5.0117E−01	5.6728E−01	3.9360E−01
Linear-Regression	4.9052E−01	6.1563E−01	5.6272E−01	5.2986E−01	5.1582E−01
ANN	3.2542E−01	5.4404E−01	4.0227E−01	6.3503E−01	3.9988E−01
RMSE					
MISMFO-MKSVR	**8.9386E**−**02**	**1.0091E**−**01**	**1.1046E**−**01**	**8.2079E**−**02**	**8.1746E**−**02**
SVR-Poly	1.1232E−01	1.1205E−01	1.3563E−01	8.2275E−02	8.6988E−02
SVR-Linear	9.0545E−02	1.0723E−01	1.1656E−01	8.8308E−02	8.8511E−02
SVR-RBF	1.1643E−01	1.4006E−01	1.3394E−01	9.2017E−02	1.1625E−01
Linear-Regression	1.5245E−01	1.4022E−01	1.1704E−01	9.2229E−02	1.0447E−01
ANN	2.4060E−01	2.3988E−01	2.2544E−01	1.7029E−01	1.3182E−01
MAE					
MISMFO-MKSVR	**4.9140E**−**02**	**7.3929E**−**02**	**7.6498E**−**02**	**3.8111E**−**02**	**3.3847E**−**02**
SVR-Poly	6.3561E−02	7.7924E−02	9.2871E−02	3.8243E−02	3.7107E−02
SVR-Linear	5.1101E−02	7.4733E−02	7.8871E−02	3.8429E−02	3.5263E−02
SVR-RBF	5.2321E−02	8.0288E−02	8.2596E−02	3.8866E−02	4.3314E−02
Linear-Regression	9.8637E−02	9.8828E−02	8.7209E−02	4.3918E−02	4.0860E−02
ANN	1.5031E−01	1.6787E−01	1.6653E−01	9.2970E−02	5.7309E−02

Optimal values are in bold.

## Conclusions and future directions

In this study, a novel variant of MFO, MISMFO, is proposed and applied to the parameter optimization of MKSVR. MISMFO initializes the moth population using Logistic chaotic mapping to improve the diversity of the initial population. Subsequently, it employs a flame mutation mechanism to perturb the flames with lower-ranked fitness values, thereby increasing the population diversity throughout the search process and enabling the algorithm to escape from local optima. Concurrently, MISMFO introduces a flame number phased reduction mechanism that strategically reduces the number of flames across iteration stages, ensuring that moths initially prioritize exploration and subsequently shift to exploitation in the later phases, effectively enhancing search efficiency. Finally, an adaptive weight mechanism is proposed to update the moths positions, allowing them to autonomously adjust their search strategies based on fitness values, thus balancing the exploration and exploitation, thereby accelerating convergence and enhancing accuracy.

On the fifteen benchmark test functions, the convergence and scalability of MISMFO are first analyzed based on three dimensions. Moreover, MISMFO is tested and compared with 13 other optimization algorithms on both the 15 classic datasets and the CEC2020 test set, and the test results are evaluated using the Bonferroni–Dunn test and the Friedman test. The results show that MISMFO achieves superior accuracy and convergence compared to existing methods. Additionally, the proposed MISMFO-MKSVR model is applied to estimate the software effort on five publicly available datasets, showing enhanced performance over competing models in addressing the software effort estimation problem. The MISMFO algorithm demonstrates superior performance in accuracy and convergence, outstripping existing methods as confirmed by comprehensive tests. It integrates four strategic approaches to optimize exploration and exploitation, enhancing quality of solutions. Applied to software effort estimation, the MISMFO-MKSVR model proves superior to other models. However, owing to its structural complexity and sensitivity to hyperparameters such as $\delta _1$ and $\delta _2$, the algorithm may pose challenges in understanding and application for practitioners.

There is still much worthwhile work to be done in the future. While the introduction of logistic chaos mapping, flame number phased reduction, flame mutation, and the adaptive weight mechanism has significantly improved performance in solving the software effort estimation problem, it has also increased algorithmic complexity. In subsequent study, we will investigate alternative methods to efficiently manage algorithmic complexity while simultaneously enhancing performance. Additionally, we plan to utilize an ensemble learning framework to estimate software effort and optimize parameters with this optimization algorithm.

## Data Availability

The datasets used and/or analysed during the current study available from the corresponding author on reasonable request.
